# *Helitron* distribution in Brassicaceae and whole Genome *Helitron* density as a character for distinguishing plant species

**DOI:** 10.1186/s12859-019-2945-8

**Published:** 2019-06-24

**Authors:** Kaining Hu, Kai Xu, Jing Wen, Bin Yi, Jinxiong Shen, Chaozhi Ma, Tingdong Fu, Yidan Ouyang, Jinxing Tu

**Affiliations:** National Key Laboratory of Crop Genetic Improvement, Huazhong Agricultural University, Wuhan, 430070 People’s Republic of China

**Keywords:** Transposable element, Plant classification, Multivariate analysis, Genomic evolution, Bioinformatics

## Abstract

**Background:**

*Helitron* is a rolling-circle DNA transposon; it plays an important role in plant evolution. However, *Helitron* distribution and contribution to evolution at the family level have not been previously investigated.

**Results:**

We developed the software easy-to-annotate *Helitron* (EAHelitron), a Unix-like command line, and used it to identify *Helitrons* in a wide range of 53 plant genomes (including 13 Brassicaceae species). We determined *Helitron* density (abundance/Mb) and visualized and examined *Helitron* distribution patterns. We identified more than 104,653 *Helitrons*, including many new *Helitron*s not predicted by other software. Whole genome *Helitron* density is independent from genome size and shows stability at the species level. Using linear discriminant analysis, de novo genomes (next-generation sequencing) were successfully classified into *Arabidopsis thaliana* groups. For most Brassicaceae species, *Helitron* density negatively correlated with gene density, and *Helitron* distribution patterns were similar to those of *A. thaliana*. They preferentially inserted into sequence around the centromere and intergenic region. We also associated 13 *Helitron* polymorphism loci with flowering-time phenotypes in 18 *A. thaliana* ecotypes.

**Conclusion:**

EAHelitron is a fast and efficient tool to identify new *Helitrons*. Whole genome *Helitron* density can be an informative character for plant classification. *Helitron* insertion polymorphism could be used in association analysis.

**Electronic supplementary material:**

The online version of this article (10.1186/s12859-019-2945-8) contains supplementary material, which is available to authorized users.

## Background

Transposons or transposable elements (TEs) are mobile DNA segments first described by McClintock in 1950 [1]. They are divided into two main classes, Class I TEs (RNA transposons or retrotransposons) that require an RNA intermediate and use a ‘copy-and-paste’ mechanism to insert their copies into new locations, and Class II elements are DNA transposons which use a ‘cut-and-paste’ mechanism to mobilize themselves without RNA intermediates [2]. *Helitrons* transpose by rolling-circle replication (RCR) with only one strand cut and are important DNA transposons (Class II) in diverse eukaryotic genomes. They were discovered by data mining the *Arabidopsis thaliana*, *Oryza sativa*, and *Caenorhabditis elegans* genomes [3]. Canonical *Helitron*s have conservative 5′-TC, CTRR-3′ (mostly CTAG-3′) termini and contain a 16–20 nt GC-rich hairpin structure located 10–15 nt upstream of the 3′ end [3, 4], which is thought to serve as a stop signal in the transposition process [5]. They have always been inserted into 5′-AT-3′ target sites and do not have terminal inverted repeats [4]. *Helitrons* can be classified as either autonomous or non-autonomous based on whether they contain the RepHel sequence, which is a protein domain homologous to the prokaryotic Rep protein involved in PCR and helicases [3].

Brassicaceae, formerly Cruciferae, is a medium-sized plant family, composed of more than 372 genera and 4060 species [6]. The family includes many important species, such as the model plant *A. thaliana* [7], the crop *Brassica rapa* [8], and *Brassica oleracea* (Cabbage) [9, 10]. Many species in this family have sequenced genomes, which are useful for *Helitron* evolution research at family level. *Helitron* length is highly variable in plants, e.g., *A. thaliana* repeat elements *AthE1* [11], *AtREP* [12], and *Basho* [13] are non-autonomous *Helitrons*, and their length ranges from 0.5–3 kb [14]. Some autonomous *Helitron*s have been found to be larger (8–15 kb in *A. thaliana*, 10–15 kb in *O. sativa,* and 5–8 kb in *C. elegans*) [3]. Maize *Helitron* length has a wide range from 202 bp to 35.9 kb [15]. In addition, some studies have shown that plant genomes have variable *Helitron* content, approximately 2% in *Arabidopsis* [3], 6.6% in maize, and 0.1–4.3% in other plants [4]. DNA transposons use a ‘cut-and-paste’ mechanism unlike the RNA transposons that use a ‘copy-and-paste’ mechanism, and are usually present in low to moderate numbers [2]. *Helitrons* are unique DNA transposons transported by RCR, a process that was confirmed by reconstructing the ancient element *Helraiser* from the bat genome [16]. However, it has also been found that *Helitron*s can excise and leave footprints, an outcome not expected from rolling-circle transposition in maize [17]. Therefore, *Helitron*s may exhibit both ‘copy-and-paste’ and ‘cut-and-paste’ modes of transposition. These reports imply that the number of *Helitrons* in the genome may be lower and steadier than RNA transposons. Therefore, *Helitron* related data may be more representative of plant genome features than RNA transposons.

*Helitrons* may express preference in terms of genomic position and have been reported to be more abundant in gene-poor regions of *Arabidopsis* [18], especially around the centromere as with other TEs [19]. However, a less ordered pattern of *Helitron* distribution was reported in rice [18]. Furthermore, it was found that the *Helitrons* of maize mainly exist in the gene-rich region rather than the gene-poor region [20]. This may be because the maize genome is larger; therefore, the density of the maize gene-rich region is similar to that of the *Arabidopsis* gene-poor region. Xiong et al. found in plant *Helitron*s amplified by RCR that the tandemly arrayed replication products mostly accumulated in the centromeres [21]. *Helitron* distribution patterns remain unclear in a wide range of plant genomes and require further research.

Similar to other transposons such as CACTA [22] and MULEs (Mutator-like elements) [23], *Helitrons* can capture gene fragments and move them around the genome [24]. It is one of the most important agents in gene evolution. *Helitrons* can change many gene functions and have been found to cause phenotypic differences by insertion into promotors leading to changes in expression patterns. A spontaneous pearly-s mutant of *Ipomoea tricolor* cv. ‘Heavenly Blue’ displays stable white flowers and is caused by an 11.5 kb *Helitron* inserted into the *DFR-B* gene for anthocyanin pigmentation [25]. In Brassicaceae, a 4.3 kb *Helitron* inserted into the *BrTT8* intron resulted in *B. rapa* with a yellow seed coat [26]. A 3.6 kb non-autonomous *Helitron* was inserted into the promoter of the determining gene for self-incompatibility in males *BnSP11–1*, which led to oilseed rape *Brassica napus* becoming self-compatible [27]. Locating these *Helitrons* is an important task in plant functional genomic research.

There are two main types of software used for searching *Helitrons*. Homology comparison software, such as CENSOR [28], RepeatMasker [29], etc., are mainly based on NCBI-BLAST [30], WU-BLAST [31] and other derivatives programs (e.g. RMBlast) comparable with Repbase [32] and other repeat sequences databases. While BLAST is not able to fully identify various *Helitrons* hairpins, similarity searches alone are not effective in identifying *Helitrons*. The other type of software, such as HelitronFinder [33] and HelSearch [18] are based on *Helitron* conserved structures. HelitronScanner identifies *Helitron* terminal structures based on a motif-extracting algorithm proposed initially in a study of natural languages [4]. It may be able to discover novel *Helitron*s but results in a high number of false positives when using the default settings [4]. With the development of next-generation sequencing (NGS) and 3rd-generation sequencing (3GS), more plant genomes have been sequenced and assembled, and a faster and easier way to annotate *Helitrons* and present annotation results is required.

In this study, we developed the software easy-to-annotate *Helitron* (EAHelitron), a rapid and easy-to-use program for computationally identifying *Helitrons*. It predicted more than 104,653 *Helitron*s in 53 genomes of different plant species (including 16 genomes from 13 Brassicaceae species) and 18 *A. thaliana* ecotype genomes. We considered whole genome *Helitron* density to be a species-specific characteristic of plants, given its potential for plant classification. We investigated the large plant family Brassicaceae in terms of *Helitron* distribution and insertion patterns. Finally, we attempted to associate flowering-time phenotypes with *Helitron* polymorphisms in 18 different *A. thaliana* ecotypes. The software and results may contribute to our knowledge of *Helitrons* and their role in plant evolution.

## Results

### Workflow of EAHelitron

**Fig. 1:**
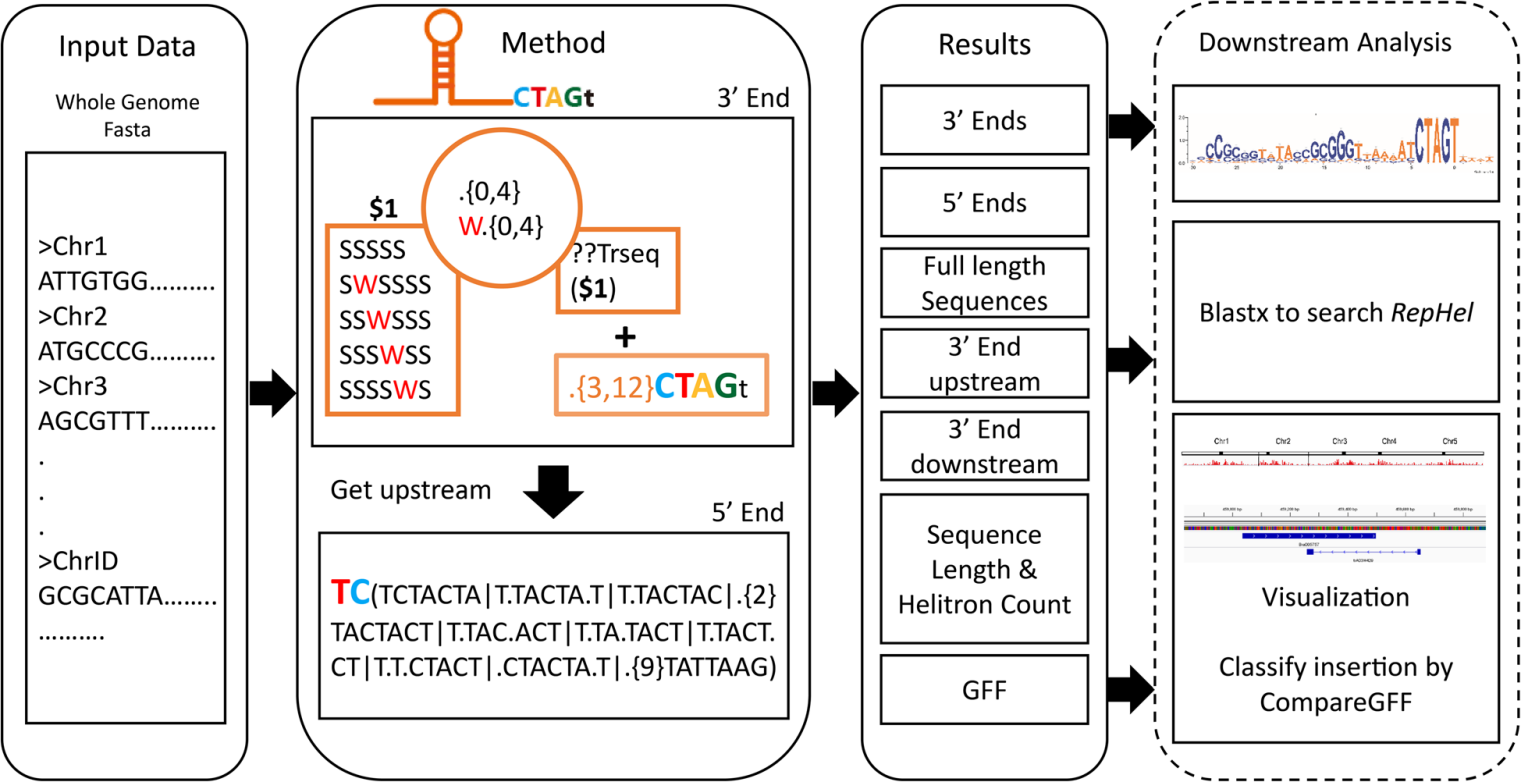
Overview of EAHelitron workflow. Left: the input data of EAHelitron. EAHelitron supports inputs of separate FASTA files or a whole genome FASTA. Middle: the method of EAHelitron. EAHelitron searches the left part of GC-rich hairpin. Next using Perl regular expressing engine’s embedded-code with TRSeq function to get the reverse complementary sequence of left part hairpin, which as the right part to complete the regular expression to continue the full-length hairpin searching. Then get the up and downstream sequences of hairpin to search 5′ TC ends and 3′ CTAG ends (S means G or C, W means A or T, ‘.’ Means A, T, G or C). Right: outputs of EAHelitron. FASTA files of ends or full length *Helitrons*, summary of *Helitron* numbers and GFF annotation

EAHelitron predicts putative *Helitrons* based on definitive features by scanning for conserved structural traits: 5′ end with TC and 3′ end with CTAG and a GC-rich hairpin loop 2–10 nt in front of the CTAG end. Using the Perl regular expression engine, the left GC-rich part of hairpin was searched by EAHelitron, followed by the capture of reverse complementary sequence of GC-rich fragment as the right part of hairpin, using our TRSeq function by an embedded-code of Perl regular expression engine. Next, the upstream and downstream sequences of hairpin were searched simultaneously using EAHelitron, to identify possible matched structure of 5′ end with TC and 3′ end with CTAG. Subsequently, such searching process was repeatedly performed by EAHelitron using the reversed complementary chromosome sequences. Finally, all records of putative *Helitrons* were printed in FASTA format including the terminal ends, 3′ upstream and downstream sequences, possible full-length *Helitron* sequences, and a general feature format (GFF) annotation file (Fig. 1).

### Comparison of EAHelitron with other software

EAHelitron supports whole genome FASTA sequences and multi-threading. Compare the time cost of *Helitrons* (4 min) searching in *Arabidopsis* TAIR10 with other software (HelitronScanner, Helsearch and RepeatMasker), EAHelitron increases the maximum speed of the prediction process by 99.3 times (38 min for HelitronScanner, 7 h for Helsearch, and 2.5 h for RepeatMasker shown in Table 1).

**Table 1:** The running time of four programs for *Helitron* identification in TAIR10

	EAHelitron	HelitronScanner	Helsearch	RepeatMasker
Threads: 1	0:04:16	0:38:15	7:03:40	2:27:34
Threads: 4	0:01:42	0:37:30	–	0:45:15

**Fig. 2:**
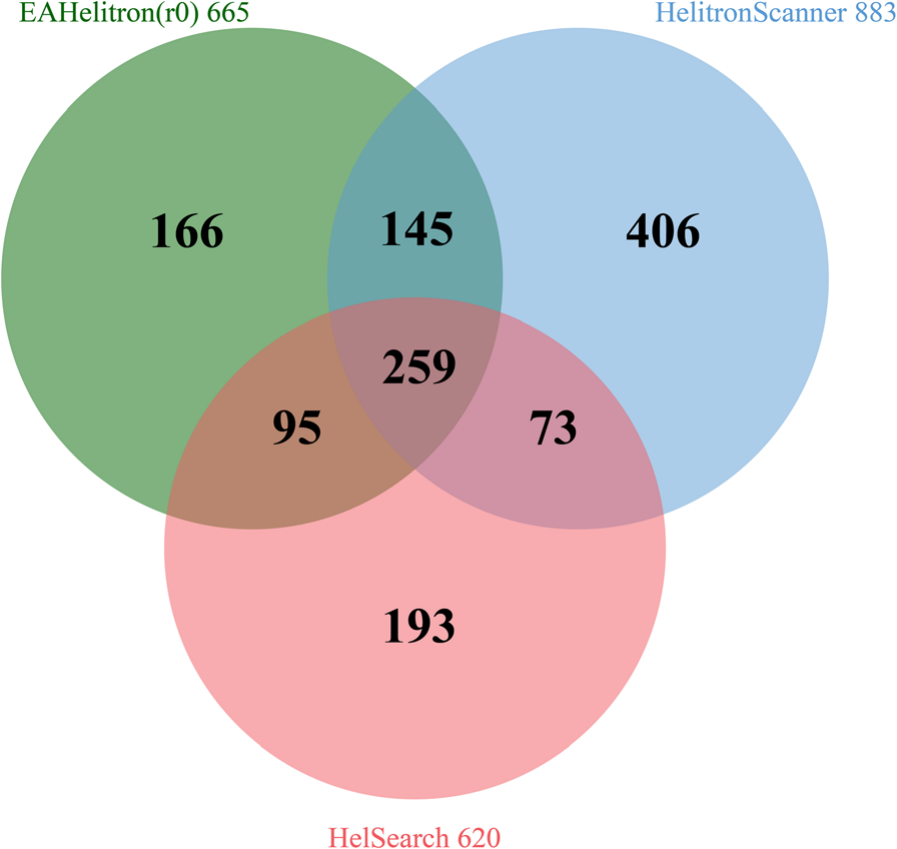
Venn diagram of predicted *Helitrons* in TAIR 10 by three programs**.** Green: EAHelitron predicts 665 *Helitrons*, including 166 uniquely records. Blue: HelitronScanner predicts 883 *Helitrons*, including 406 uniquely records. Red: HelSearch predicts 620 *Helitrons*, including 193 uniquely records. Three software share 259 *Helitron* records. EAHelitron shares 354 and 404 *Helitrons* with HelSearch and HelitronScanner separately. In total, 499 EAHelitron-predicted *Helitrons* (75% of 665) are supported by HelSearch or HelitronScanner

We ran EAHelitron against genome sequences of TAIR10 at the default 3′ terminal fuzzy level and identified 665 *Helitrons*. Comparing these results with those of former programs, we found that 75.0% of the EAHelitron-predicted *Helitrons* (499/665) were supported by HelSearch or HelitronScanner (Fig. 2, Additional file 2: Table S1). In silico verification of EAHelitron-predicted *Helitron*s through the study of in 18 different *A. thaliana* ecotypes showed that at least 508 *Helitron*s were active in transposition in these ecotypes (Additional file 2: Table S2), including at least 41 *Helitron*-insertion polymorphisms of the 166 (24.7%) *Helitron*s uniquely predicted by EAHelitron in TAIR10 (Additional file 2: Table S1 and S2). This indicates that EAHelitron has the ability to find genuine new *Helitron*s.

To estimate the false positive rates (FPR) of these programs, we predicted *Helitron*s in 100 randomly reconstructed genomic sequences of *Arabidopsis* using EAHelitron, HelSearch and HelitronScanner [18]. HelitronScanner had the highest FPR under the default settings (32.67%, Additional file 2: Table S3), and EAHelitron showed lower FPR of 5.91% (Additional file 2: Table S3). HelSearch operates by only counting those occurrences with more than one copy; therefore, no false positive *Helitrons* were identified in these random genomes (not listed). However, the omission of one-copy *Helitrons* in this application can be a problem. EAHelitron provides outputs in the form of full length *Helitron*s, flanking sequences, and support-to-output GFF3 files, similar to RepeatMasker [29], which are easy for presenting *Helitron*s in genome visualization software (389 of EAHelitron-predicted *Helitrons* were supported by RepeatMasker, Additional file 1: Figure S1), such as IGV [34], GBrowse [35], and JBrowse [36]. Considering the time cost, support of whole genome automatic annotation, acceptable FPR, convenience of downstream analysis, and visualization, we used EAHelitron to identify *Helitrons* in subsequent analysis of plant genomes.

### *Helitron* identification in 53 plant genomes

**Fig. 3:**
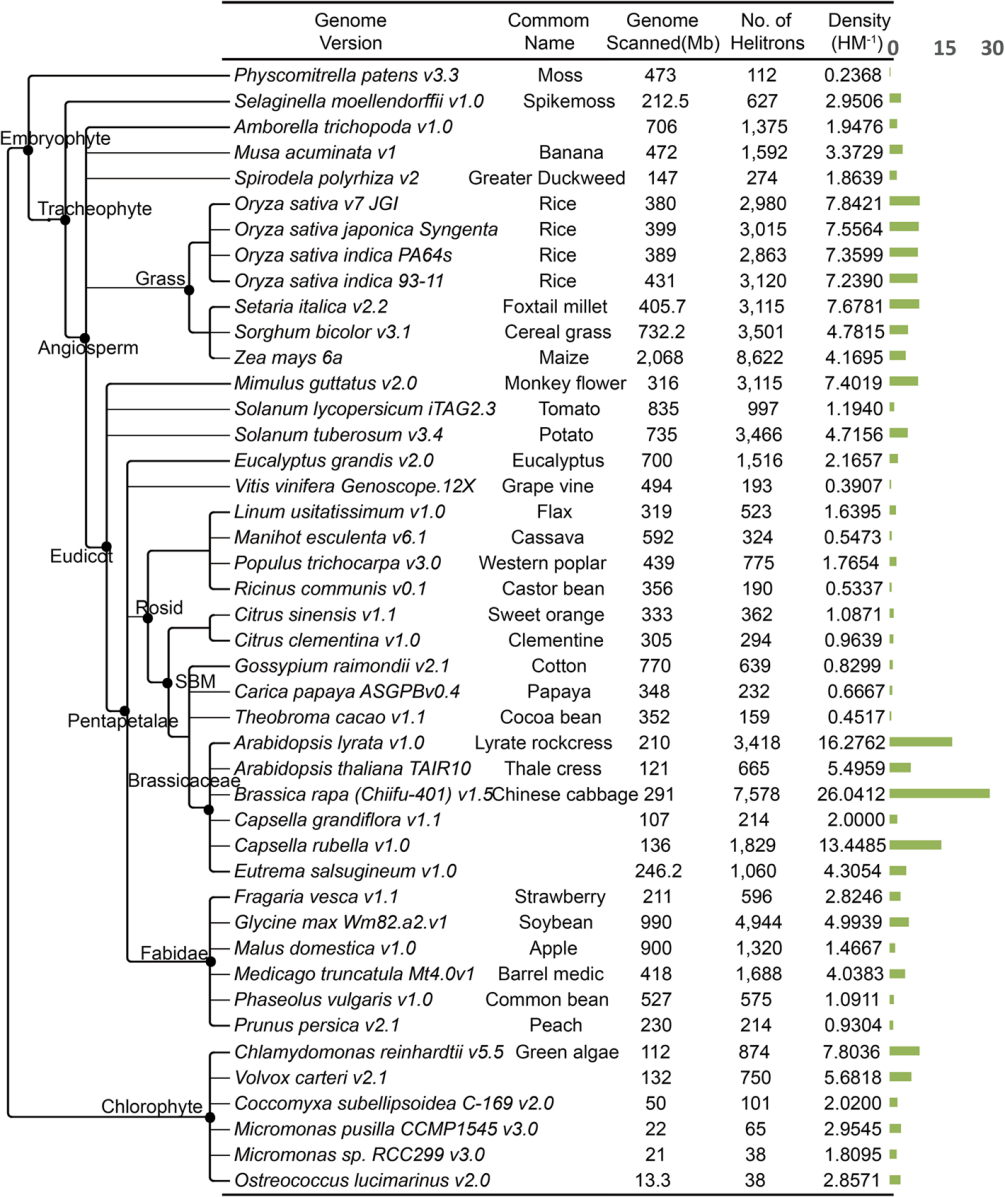
Genome and *Helitron* information of 44 plant genomes. Left phylogenetic tree is constructed based on Phytozome V11 and APG. Right green blocks represent *Helitron* density. A plant family could have a quite different counts of *Helitron* and *Helitron* density, like Brassicaceae

Using EAHelitron, we identified 104,653 *Helitrons* in 53 published plant genomes, including a wide range of monocots and eudicots (Additional file 3: Table S4). The 5′ terminal ends of *Helitrons* are less conserved than 3′ ends [4]. In addition, a *Helitron* may have a single 3′ end but multiple 5′ termini [21], which results in difficulties in predicting *Helitron* length. It makes genome content of *Helitron* that, based on *Helitron* length, would not be accurate to describe a genome character. Here, we used *Helitron* density, defined by the number of 3′ termini of *Helitrons* divided by the genome size, which is potentially a more accurate genomic characteristic than the proportion of *Helitron* sequence length in the genome. The phylogenetic relationship, based on APG [37] and Phytozome 11, genome sizes, and *Helitron* numbers, *Helitron* densities of 53 plant genomes were summarized in Fig. 3 and Table 2. The number of *Helitrons* varied dramatically among these plant genomes. *B. napus* contained the largest number of *Helitrons* (13,968), while in *Ostreococcus lucimarinus* and *Micromonas* sp. RCC299, only 38 *Helitrons* were detected in each of the genomes representing the minimum number of *Helitron*s. Notably, sibling species may have divergent *Helitron* densities, even though they belong to the same family (Fig. 3). For example, a 3-fold difference in *Helitron* density between *A. thaliana* and *A. lyrata* (5.5 and 16.3, respectively) was detected, indicating significant variation in either *Helitron* counts or *Helitron* densities in *Arabidopsis* genus. So, either *Helitron* counts or *Helitron* densities (0.2368–26.0412) greatly varied in these plants.

**Table 2:** Summrization of related information for *Helitrons* identified in Brassicaceae

Taxon	Genome Size (M)	*Helitron*	HM^−1^	Gene zone	CDS	Intron or UTR	Intergenic region
*Brassica rapa* v1.5	291.7	7578	25.9788	394	41	353	7184
*Arabidopsis lyrata*	210.2	3418	16.2607	101	24	77	3317
*Brassica napus* v5	864.5	13,968	16.1573	807	84	723	13,161
*Brassica oleracea* TO1000 v2.1	498.9	6979	13.9888	262	53	209	6717
*Brassica oleracea* A2 v1.0	391.4	5392	13.7762	153	25	128	5239
*Capsella rubella*	134.8	1829	13.5683	65	10	55	1764
*Camelina sativa*	648.7	5827	8.9826	248	55	193	5579
*Arabidopsis thliana*	121	665	5.4959	70	18	52	595
*Aethionema arabicum*	200.2	1028	5.1349	72	37	35	956
*Thellungiella salsuginea* v2	233.7	1032	4.4159	–	–	–	–
*Thellungiella halophila 173 (Esa)*	246.2	1060	4.3054	40	20	20	1020
*Leavenworthia alabamica*	174.4	450	2.5802	55	30	25	395
*Sisymbrium irio*	260.5	603	2.3148	41	22	19	562
*Capsella grandiflora* v1.1	112.3	214	1.9056	17	9	8	207
*Thellungiella parvula* v8	123.6	202	1.6343	45	18	27	157
*Schrenkiella parvula*	140	223	1.5929	–	–	–	–

“-” Lack of GTF

To study the *Helitron* features in different sequenced genomes from one species, we compared the characteristic of *Helitrons* in different sequenced genomes of seven species (*Oryza sativa japonica*, *Oryza sativa indica*, *Eutrema salsugineum* or formerly *Thellungiella salsuginea*, *Schrenkiella parvula* or formerly *Thellungiella parvula*, *Brassica oleracea*, *Arabidopsis thaliana* and *Zea mays*, Table 3). The results showed that although the genome size and *Helitron* numbers varied in different varieties or ecotypes of the same species, the densities of *Helitrons* remained relatively stable. In rice, the genome size for two *indica* varieties PA64s and 93–11 were 389 M and 431 M, respectively, with a standard deviation (SD) of 29.70 and coefficient of variation (CV) of 7.24%. Also, the number of *Helitrons* were 2863 for PA64s and 3120 for 93–11 (SD = 181.73, CV = 6.07%). However, the *Helitron* densities were 7.36 for PA64s and 7.24 for 93–11, which was is a constant value in rice species (SD = 0.086, CV = 1.17%). Similarly, in *B. oleracea* A2 v1.1 and *B. oleracea* TO1000 v2.1, their genome size (391 M and 498 M, respectively, SD = 75.66, CV = 17.02%) and *Helitron* number (5392 and 6979, respectively, SD = 1122, CV = 18.14%) were different, but their *Helitron* densities were similar (~ 13.90 *Helitrons* per Mb, SD = 0.16, CV = 1.14%). And compression of two version of *Thellungiella salsuginea* genomes showed that, *Thellungiella salsuginea* and *Eutrema salsugineum* (formerly *Thellungiella halophila*, which finally were determined to be *Thellungiella salsuginea*) had steadier *Helitron* density (~ 4.36 *Helitrons* per Mb, SD = 0.055, CV = 1.27%) than genome size (233.7 M and 246.2 M, respectively, SD = 6.25, CV = 2.60%). Therefore, *Helitron* density may be regarded as a stable genomic characteristic.

**Table 3:** Linear discriminant analysis (LDA) of 36 plant genome samples

Training label	Genome	Genome Size (M)	*Helitron*	HM^−1^	LDA predicted
*Osa* japonica	*Oryza sativa japonica* IRGSP v7_JGI	380	2980	7.8421	*Osa* japonica
*Osa* japonica	*Oryza sativa japonica* Syngenta	399	3015	7.5564	*Osa* japonica
*Osa* indica	*Oryza sativa indica* PA64s	389	2863	7.3599	*Osa* indica
*Osa* indica	*Oryza sativa indica* 93–11	431	3120	7.239	*Osa* indica
*Ath*	*A. thaliana* Col-0	121	665	5.4959	*Ath*
*Ath*	*A. thaliana* Can-0	119.3	590	4.9455	*Ath*
*Ath*	*A. thaliana* Zu-0	119.7	590	4.929	*Ath*
*Ath*	*A. thaliana* Po-0	120.5	593	4.9212	*Ath*
*Ath*	*A. thaliana* Hi-0	120.3	592	4.921	*Ath*
*Ath*	*A. thaliana* Oy-0	119.5	575	4.8117	*Ath*
*Ath*	*A. thaliana* Wu-0	119.7	572	4.7786	*Ath*
*Ath*	*A. thaliana* Sf-2	119.6	567	4.7408	*Ath*
*Ath*	*A. thaliana* Ct-1	119.6	567	4.7408	*Ath*
*Ath*	*A. thaliana* Mt-0	119.5	565	4.728	*Ath*
*Ath*	*A. thaliana* Edi-0	119.8	564	4.7078	*Ath*
*Ath*	*A. thaliana* Tsu-0	119.6	559	4.6739	*Ath*
*Ath*	*A. thaliana* Bur-0	119.7	556	4.6449	*Ath*
*Ath*	*A. thaliana* Rsch-4	119.8	554	4.6244	*Ath*
*Ath*	*A. thaliana* Ler-0	119.7	552	4.6115	*Ath*
*Ath*	*A. thaliana* Ws-0	119.8	547	4.5659	*Ath*
*Ath*	*A. thaliana* Wil-2	119.5	543	4.5439	*Ath*
*Ath*	*A. thaliana* Kn-0	119.7	542	4.528	*Ath*
*Tsa*	*Eutrema salsugineum* v1.0	246.2	1060	4.3054	*Tsa*
*Tsa*	*Thellungiella salsuginea* v2	233.7	1032	4.4159	*Tsa*
*Tpa*	*Thellungiella parvula* v8	123.6	202	1.6343	*Tpa*
*Tpa*	*Schrenkiella parvula*	140	223	1.5929	*Tpa*
*Bol*	*Brassica oleracea* A2 v1.1	391	5392	13.7903	*Bol*
*Bol*	*Brassica oleracea* TO1000 v2.1	498	6979	14.0141	*Bol*
*Zma*	*Zm B73* V4.0	2134	8274	3.8765	*Zma*
*Zma*	*Zm CML247* V1.1	2197	8791	3.9996	*Zma*
*Zma*	*Zm EP1* V1.0	2455	8481	3.4542	*Zma*
*Zma*	*Zm F7* V1.0	2392	8602	3.5949	*Zma*
*Zma*	*Zm Mo17* V1.0	2182	8602	3.9412	*Zma*
*Zma*	*Zm W22* V2.0	2133	8132	3.8109	*Zma*
	**Denovo_genome_L**	121.1	640	5.2849	***Ath***
	**Denovo_genome_X**	120.2	643	5.3494	***Ath***
Correct Rate					36/36 = 100%

De novo plant genomes are bolded

To further estimate the relationship between genome size, *Helitron* number, and *Helitron* density, we calculated the Pearson’s product-moment correlation in 53 plant genomes (Table 4, Additional file 1: Figure S2,). The results suggested that *Helitron* number was significantly positively correlated with genome size and *Helitron* density (r1 = 0.52, p1 = 7.23E-05; r2 = 0.71, p2 = 2.60E-09); however, *Helitron* density may not be correlated with genome size (*p* = 0.73). Therefore, *Helitrons* contributed to the size changes in plant genomes, whereas *Helitron* density and genome size are independent of each other, we can use *Helitron* density as a genome character together with genome size in the next experiments.

**Table 4:** Pearson’s product-moment correlation with *Helitron* number, *Helitron* density and genome size of 53 plant genomes (1000 bootstrap replicates)

	r	r 95% confidence interval		r bootstrap 95% BCa		p	p bootstrap 95% BCa	
*Helitrons* vs Genome size	0.5176	0.2875	0.6912	0.2280	0.7429	7.23E-05	0.0000	0.1671
*Helitrons* vs *Helitron* Density	0.7102	0.5444	0.8226	0.3215	0.8243	2.60E-09	0.0000	0.0056
*Helitron* Density vs Genome size	−0.0492	−0.3153	0.2241	−0.1844	0.2528	0.7267	0.2871	0.9980

Considering the stability of *Helitron* density at the species level, it might be regarded as a species-specific characteristic for use in classification. To validate the efficacy of using *Helitron* density to identify species, we performed the LDA using seven genomes with at least two sequence variants (Table 3). In total, 34 genomes (including 18 *A. thaliana*) were used to train the model in R with *Helitron* density and genome size. Next, we added the *Helitron* information from two de novo assembled genomes of *A. thaliana* mutants, Denovo_genome_L (CS852557, N50: 5064, Scaffolds: 3350) and Denovo_genome_X (SALK_015201, N50: 25,619, Scaffolds: 9888) to these data, and then predicted which species groups they belong to. LDA predicted all of these 36 samples correctly (100%), including successfully identifying the two de novo samples to the *A. thaliana* group from six other species groups (Table 3, Additional file 1: Figure S3). This result indicated that EAHelitron can count the *Helitrons* of NGS de novo genome drafts successfully, and that *Helitron* density is informative as a species-specific characteristic in plant genomes and could be applied to expediate plant identification.

### Identification of *Helitron*s in Brassicaceae

Many Brassicaceae species genomes are sequenced and are informative for *Helitron* evolution research. There were 49,213 *Helitron*s were predicted from 16 Brassicaceae genomes, showing a wide range of diversity in genome size, *Helitron* count, and *Helitron* density (Table 2, Additional file 1: Figure S4). Of these genomes, *B. napus* had the largest genome size and *Helitron* counts (864.5 M and 13,968, respectively). *Capsella grandiflora* had the smallest genome (112.3 M) and *T. parvula* v8 had the least number of *Helitron*s (202)*.* The *Helitron* density reached a maximum of 25.98 in *B. rapa*, whereas *T. parvula* had the lowest *Helitron* density of 1.59. Most of *Helitron*s in Brassicaceae were non-autonomous, only 1.6–18.49% were autonomous (6.5% in average, Additional file 2: Table S5). Also, RepHel percentage was not correlated with *Helitron* density or *Helitron* number (p1 = 0.21, p2 = 0.24, Additional file 2: Table S5), which means autonomous *Helitron* counts were not correlated with the total *Helitron* number in host genomes of Brassicaceae. *B. napus* (genome AnAnCnCn) was formed by recent allopolyploidy (7500 to 12,500 years ago) between ancestors of *B. oleracea* (CoCo) and *B. rapa* (ArAr) [38]. We found that the *Helitron* density of subgenomes in *B. napus* decreased relative to the ancestor genomes of *B. oleracea* and *B. rapa*. In addition, the subgenome of An had higher *Helitron* density relative to the Cn subgenome in *B. napus* (An: 7056/314.2 = 22.4570 < Ar: 25.9788, Cn: 6721/525.8 = 12.7824 < Co: 13.9888 or 13.7762, AnCn: 16.1573 < ArCo: 18.4126 or 18.9870). This inferred that allopolyploidy may affect the density of *Helitrons* during evolution.

### *Helitrons* evolution in Brassicaceae

We constructed a dendrogram of 15 *Brassicaceae* genomes based on genome size and *Helitron* density with hierarchical clustering (Additional file 1: Figure S5a). This was compared with known phylogenetic trees, one based on a reconstruction using the ancestral Brassicaceae karyotype genome [39] (Additional file 1: Figure S5b), and the other based on sequences of nuclear ribosomal ITS-1, 5.8S ribosomal RNA, and ITS-2 region [40] (Additional file 1: Figure S5c). The *Helitron* density related dendrogram had a similar topological structure to these two known phylogenetic trees, indicating that *Helitron* density, which may contain the history of the transposon replications and genome size expanding, e.g. whole genome duplication (WGD), is informative in terms of species evolution.

We investigated the evolutionary process of *Helitron*s in eight sibling genomes in Brassicaceae (Ath, Aly, Cru, Tpa, Bol v1, Bol v2, Bra, and Bna), and upstream 1kbp sequences of 3′ termini were chosen to search for conserved sequences showing highly similarity (Additional file 2: Table S6). Although the proportion of conserved *Helitron*s (evalue <1e-5, qcov > 55, s_end > 950; length of upstream sequences of 3′ termini matched larger than 55 bp) was consistent with the phylogenetic relationship between the species, the number of conserved *Helitron*s remained at a rather low level. The divergence time of *A. lyrata* and *A. thaliana* was about 10 to 12 Mya, with approximately 90% of syntenic regions found between the two genomes. It was found that all 32,670 *A. lyrata* protein-coding genes were homologous to the 27,025 (98.7%) genes in *A. thaliana* [41]. However, only 12.4 to 22.7% of *Helitron*s were conserved between the two genomes showing homology with each other (Additional file 2: Table S6). Similarly, *B. oleracea* and *B. rapa* diverged about 4.6 Mya. A total of 66.5% (34,237 genes) of *B. oleracea* genes and 74.9% (34,324) of *B. rapa* genes were regarded as homologous [9], whereas they only shared 50.05 to 52.60% of homologous *Helitron*s. The proportion of conserved *Helitrons* between Camelineae (Ath, Aly and Cru) and Calepineae (Tpa, Bra, Bol and Bna), which diverged around 27 Mya [39], reduced to less than 1%. These results suggest that *Helitrons* evolved much quickly than protein-coding genes, and they were likely to originate in the ancestral species but diverge or disappear in some of the lineages during the evolution. We also found that a large proportion of *Helitrons* in Brassicaceae, from 35.75% in *Tpa* to 80.63% in *Aly*, were multiple copies, with an average ratio of 65.72% being multi-copy *Helitrons* (Additional file 2: Table S6). This suggested that *Helitrons* were inclined to duplicate themselves in host genomes during the evolution, but still have some *Helitrons* remained in single copy.

### *Helitron* distributions in Brassicaceae

**Fig. 4:**
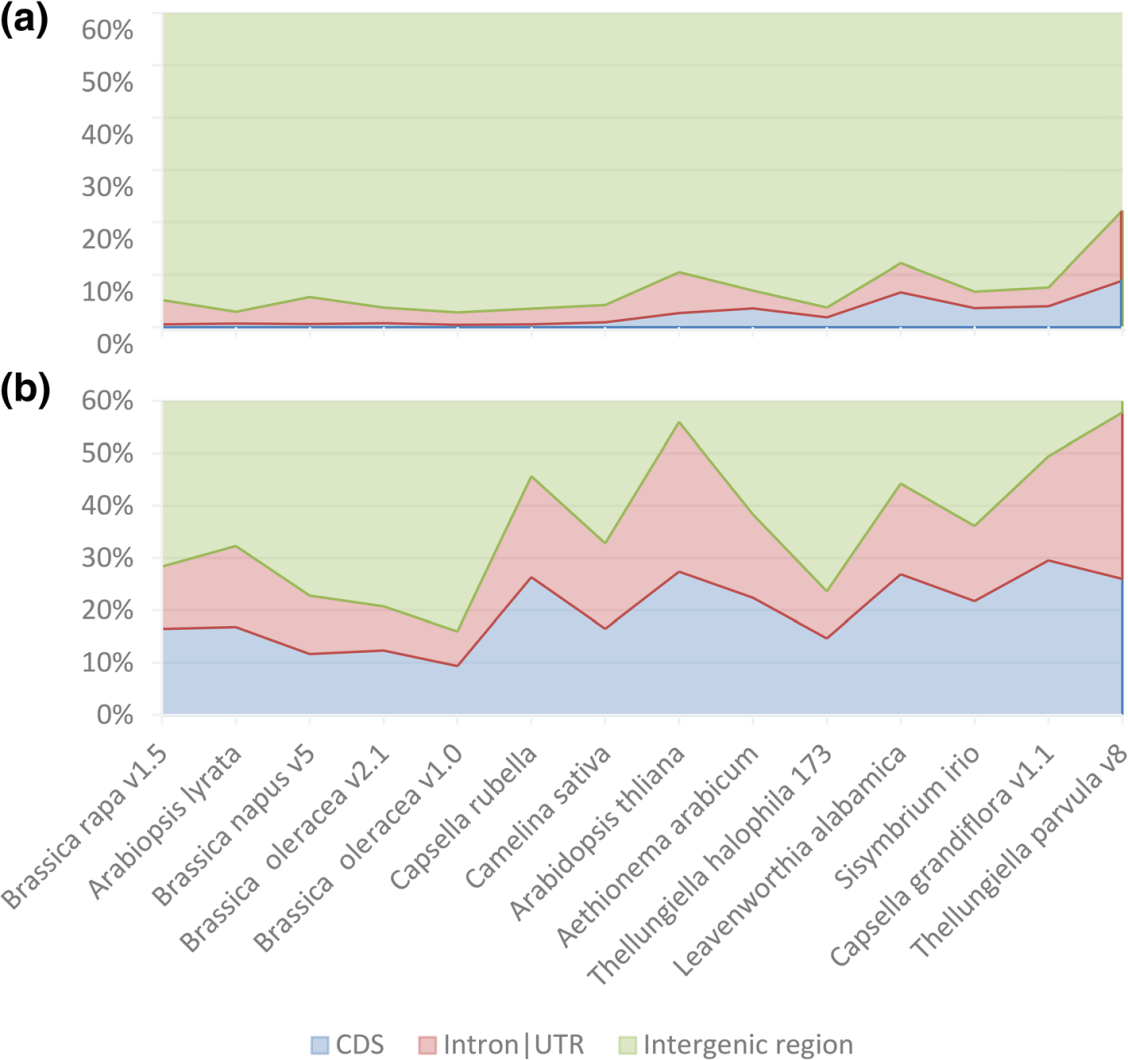
Percent of *Helitron*-insertion types. Hidden the rest 40% intergenic region percent. (**a**) *Helitron* insertion percentage accumulation map, (**b**) percentage accumulation map of CDS, Intron/UTR and intergenic region length with whole genome. *Helitron* insertion are not random (Chi-squared Test, p < 0.0001)

We further analyzed *Helitron* insertion sites using CompareGFF script. The positions of all *Helitrons* were clustered into three types: in exon, in intron or untranslated regions (UTR), and in intergenic regions (see examples in IGV in Additional file 1: Figure S6). Among these Brassicaceae genomes, *T. parvula* had the highest gene zone (exon, intron and UTR) insertion rate (22.2%), whereas *B. oleracea A2* v1.0 had the lowest *Helitron* insertion rate (2.8%). The average rate was 7% (Table 2, Fig. 4a). The Chi-square test of *Helitron* insertion rate (Fig. 4a) with genome components rate (Fig. 4b) showed that, *Helitron*s were not distributed randomly in all tested genomes (*p* < 0.0001). Most *Helitrons* were inserted in the intergenic region (77.8 to 97.2%, 93.3% average). In general, those *Helitrons* inserted in the gene zone were mostly found in UTR or introns (4.5%) rather than in CDS (2.6%) (Fig. 4a, Table 2).

**Fig. 5:**
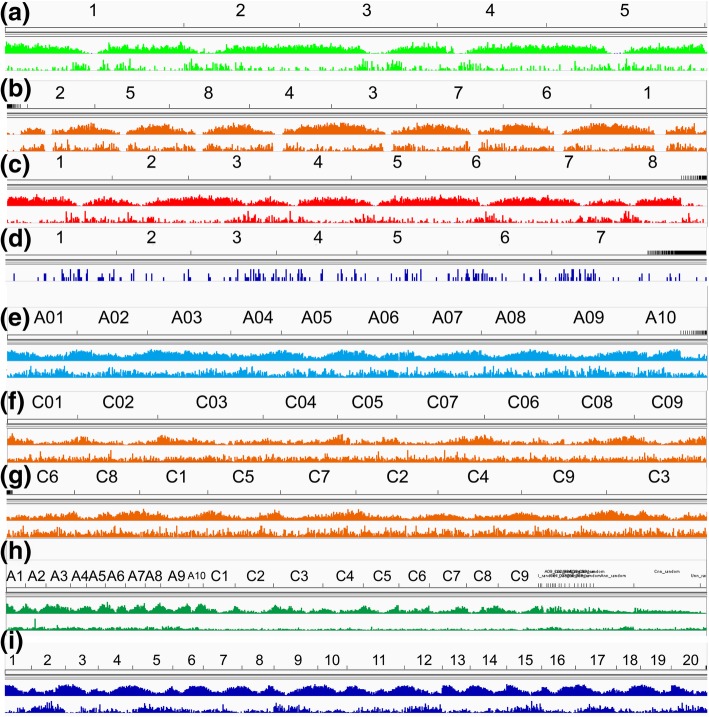
Gene and *Helitron* distribution of nine Brassicaceae genomes. First row is chromosome, middle row is gene distribution, and last row is *Helitron* distribution. (**a**) Ath, (**b**) Aly, (**c**) Cru, (**d**) Tpa (lack of GTF), (**e**) Bra, (f) Bol v1, (**g**) Bol v2, (**h**) Bna, (**i**) Csa. Most of Brassicaceae *Helitron*s prefer to locate around centromeres and lack gene region. Sliding window analysis (window = 1 Mbp, step = 500 kbp) and correlation analysis show that, most of these genomes, gene densities are high negatively related with *Helitron* density (−0.707 < r < −0.315, p < 0.001, Table S8, Additional file 2)

The relationship between gene density and *Helitron* density was also investigated, and an overview of the *Helitron* distribution of nine genomes (Ath, Aly, Cru, Tpa, Bra, Bol v1, Bol v2, Bna and Csa) on the chromosome were shown on the IGV (Fig. 5). Sliding window and correlation analyses suggested that in most of these genomes (5/8), local gene densities of windows were highly negatively related to local *Helitron* densities (− 0.707 < r < − 0.315, *p* < 0.001, Additional file 2: Table S7). Two species (*A. lyrata* and *B. napus*) were found to be slightly positively correlated (r1 = 0.130, p1 < 0.05, r2 = 0.234, p2 < 0.01, Additional file 2: Table S7). *B. oleracea Helitron* density and gene density were not correlated significantly (*p* > 0.05) These results suggested that *Helitron*s mostly preferred low-density gene areas in Brassicaceae, and this was in accordance with previously research that suggested that most *Helitron*s were located in low gene density areas especially around the centromeres in *Arabidopsis* [18].

### Analyses of functions of *Helitron*-inserted genes in Brassicaceae

**Fig. 6:**
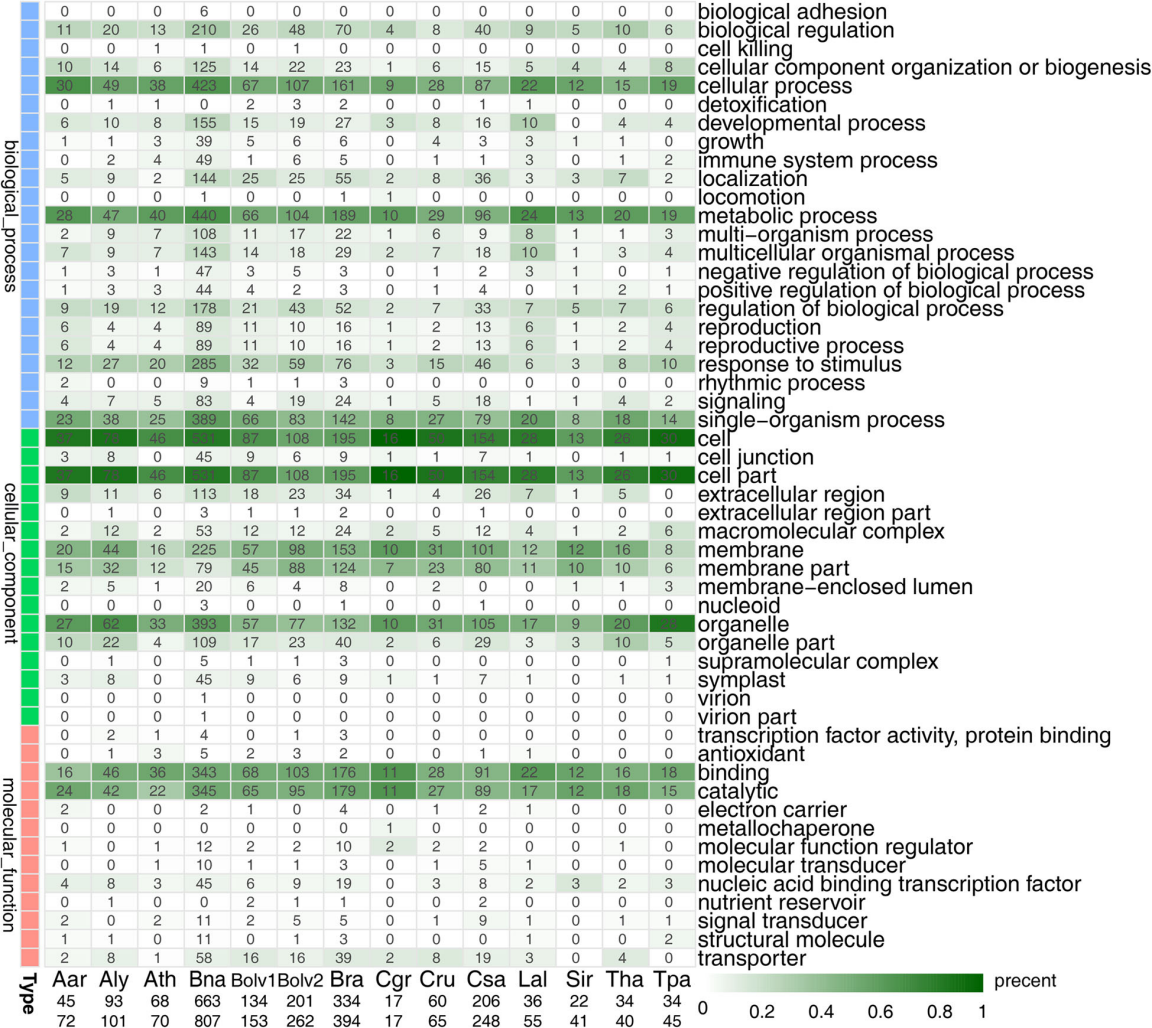
GO terms percentage heatmap of *Helitron*-inserted genes of Brassicaceae. X-axis number means annotated gene number and all *Helitron*-inserted-gene-zone (5′-UTR to 3′-UTR) number of this genome. Legend of green means gene counts percentage of all annotated genes in the GO term. These Brassicaceae genomes have similar percentage in some dark green GO terms, e.g. biological regulation, reproduction, response to stimulus, membrane, catalytic

A total of 2370 *Helitron-*inserted genes were identified in Brassicaceae (Additional file 4: Table S8). The GO terms heatmap showed that the functions of these *Helitron-*inserted genes exhibited some similar patterns, such as biological regulation, localization, metabolic process, multicellular organismal process, reproduction, and response to stimulus in biological process categories (BP), binding, catalytic, transporter, and nucleic acid binding transcription factor in molecular function categories (MF), and cell, membrane, organelle, and symplast in cellular component categories (CC) (Fig. 6).

Four well-annotated genomes (*A. thaliana*, *B. rapa*, *B. oleracea* v1, and *B. napus*) in GO terms or KEGG pathways were used for further enrichment analysis (all annotated genes were used as background). The significantly enriched results are listed in Additional file 5: Table S9 (*P* < 0.001, corrected *P* < 0.1 and hit genes > 2). In *Arabidopsis*, *Helitron-*inserted genes were likely to be enriched in terms of triplet codon-amino acid adaptor activity (GO: 0030533), binding (GO: 0005488), and other items in the MF category. *Helitron-*inserted genes in *B. rapa* were significantly enriched in terms of transmembrane transport (GO: 0055085, BP), xanthophyll metabolic process (GO: 0016122, BP), inorganic anion transport (GO: 0015698, BP), water transmembrane transporter activity (GO: 0005372, MF), lipase activity (GO: 0016298, MF), and others. *B. oleracea* v1 genome *Helitron*-inserted genes were enriched in terms of drug transport (GO: 0015893, BP), sexual reproduction (GO: 0019953, BP), transmembrane transporter activity (GO: 0022857, MF), antiporter activity (GO: 0015297, MF), and others (Additional file 5: Table S9). *B. napus Helitron-*inserted genes were enriched in terms of response to wounding (GO: 0009611, BP), suberin biosynthetic process (GO: 0010345, BP), cell periphery (GO: 0071944, CC), long-chain-fatty-acyl-CoA reductase activity (GO: 0050062, MF), carbon-oxygen lyase activity, acting on phosphates (GO: 0016838, MF), terpene synthase activity (GO: 0010333, MF), and others. The KEGG pathway enrichment showed that *A. thaliana* was enriched in Phenylpropanoid biosynthesis (map00940), and *B. oleracea* was enriched in cutin, suberine and wax biosynthesis (map00073) and lipid metabolism. However, *B. rapa* and *B. napus* were not significantly enriched in any pathways in these tests (Additional file 5: Table S9).

### *Helitron* distributions in different ecotypes of *A. thaliana*

In *Arabidopsis*, the numbers of *Helitrons* in 18 ecotypes (Additional file 1: Figure S7) varied from 542 to 665 (average 572, SD = 27.7, Table 5), with an average density of 4.77 *Helitrons* per Mb (SD = 0.21, Table 5). Ecotype Kn-0 from Kaunas, Lithuania had the least number of *Helitron*s (542), while the Col-0 ecotype from USA has the largest number of *Helitron*s (665). Of the 665 predicted *Helitrons* in Col-0, 70 *Helitrons* had been inserted in gene regions; 18 of them were located in CDS (Table 6), and 52 were located in introns or UTR (Additional file 2: Table S10). According to the TAIR10 annotation, three *Helitrons* were inserted in CDS genes (AT1G62840, AT4G11700, and AT5G66580) of unknown function (Table 6).

**Table 5:** The information of origin, type of flowering-time and *Helitron* counts from 18 *A. thaliana* ecotypes

Rank	Accession	Country	Origin	Stock	Unique LOC	M	*Helitrons*	HM^−1^	Flowering-time
1	Col-0	USA	Columbia	CS22625	1	121	665	5.4959	**intermediate**
2	Can-0	Spain	Canary Islands	CS6660	14	119.3	590	4.9455	**late**
3	Zu-0	Switzerland	Zurich	CS6902	8	119.7	590	4.9290	**late**
4	Po-0	Germany	Poppelsdorf	CS6839	3	120.5	593	4.9212	intermediate
5	Hi-0	Netherlands	Hilversum	CS6736	3	120.3	592	4.9210	intermediate
6	Oy-0	Norway	Oystese	CS6824	5	119.5	575	4.8117	**intermediate**
7	Wu-0	Germany	Wurzburg	CS6897	4	119.7	572	4.7786	intermediate
8	Sf-2	Spain	San Feliu	CS6857	11	119.6	567	4.7408	late
9	Ct-1	Italy	Catania	CS6674	5	119.6	567	4.7408	**intermediate**
10	Mt-0	Libya	Martuba/Cyrenaika	CS1380	4	119.5	565	4.7280	**intermediate**
11	Edi-0	UK	Edinburgh	CS6688	10	119.8	564	4.7078	late
12	Tsu-0	Japan	Tsushima	CS6874	5	119.6	559	4.6739	intermediate
13	Bur-0	Ireland	Burren	CS6643	6	119.7	556	4.6449	**intermediate**
14	Rsch-4	Russia	Rschew/Starize	CS6850	3	119.8	554	4.6244	intermediate
15	Ler-0	Poland	Ler	CS20	7	119.7	552	4.6115	intermediate
16	Ws-0	Russia	Wassilewskija	CS6891	9	119.8	547	4.5659	**late**
17	Wil-2	Russia	Wilna/Litvanian	CS6889	5	119.5	543	4.5439	intermediate
18	Kn-0	Lithuania	Kaunas	CS6762	6	119.7	542	4.5280	intermediate

Flowering-time types obtained from TAIR official annotation are bolded

**Table 6:** Predicted CDS-inserted genes by *Helitrons* in *A. thaliana* (TAIR10)

*Helitron* ID	TAIR ID	Descriptions
tr1H72	AT1G12160.1	Flavin-binding monooxygenase family protein
tr1H55	AT1G33520.1	D111/G-patch domain-containing protein; modifier of snc1, 2 (MOS2)
1H74	AT1G62840.1	Protein of unknown function (DUF1442)
tr1H8	AT1G64060.1	respiratory burst oxidase protein F (RBOH F);
2H52	AT2G28840.1	XB3 ortholog 1 in *Arabidopsis thaliana* (XBAT31)
tr2H6	AT2G40100.1	light harvesting complex photosystem II (LHCB4.3)
2H59	AT2G46770.1	NAC (No Apical Meristem) domain transcriptional regulator superfamily protein; EMBRYO DEFECTIVE 2301 (EMB2301)
tr3H75	AT3G04470.1	Ankyrin repeat family protein
tr3H54	AT3G28740.1	Cytochrome P450 superfamily protein; CYP81D1
tr4H30	AT4G11700.1	Protein of unknown function (DUF626)
tr4H24	AT4G16630.1	DEA(D/H)-box RNA helicase family protein
tr4H8	AT4G33020.1	ZIP metal ion transporter family; ZIP9
tr4H7	AT4G34430.1	DNA-binding family protein; CHB3
tr4H5	AT4G37150.1	methyl esterase 9 (MES9)
tr5H66	AT5G07220.1	BCL-2-associated athanogene 3 (BAG3)
tr5H12	AT5G46530.1	AWPM-19-like family protein
5H74	AT5G66580.1	unknown protein
PtH1	ATCG00700.1	photosystem II reaction center protein N (PSBN)

Bolded genes are unknown function

**Table 7:** Paired rules of *Helitron*-inserted LOCs that associated with flowering-time types in *A. thaliana* ecotypes

Left rules	Right rules	support	confidence	lift	ID	Nearest Gene
LOC006 = 0	FT = late	0.111111	1	3.6	LOC006	AT1G04425
LOC006 = 1	FT = intermediate	0.722222	0.8125	1.125	LOC006	AT1G04425
LOC008 = 0	FT = late	0.111111	1	3.6	LOC008	AT1G07450
LOC008 = 1	FT = intermediate	0.722222	0.8125	1.125	LOC008	AT1G07450
LOC118 = 0	FT = intermediate	0.722222	0.8125	1.125	LOC118	AT1G72510
LOC118 = 1	FT = late	0.111111	1	3.6	LOC118	AT1G72510
LOC131 = 0	FT = intermediate	0.722222	0.8125	1.125	LOC131	AT2G01700
LOC131 = 1	FT = late	0.111111	1	3.6	LOC131	AT2G01700
LOC137 = 0	FT = late	0.111111	1	3.6	LOC137	AT2G03990
LOC137 = 1	FT = intermediate	0.722222	0.8125	1.125	LOC137	AT2G03990
LOC241 = 0	FT = intermediate	0.722222	0.8125	1.125	LOC241	AT3G10970
LOC241 = 1	FT = late	0.111111	1	3.6	LOC241	AT3G10970
LOC256 = 0	FT = late	0.111111	1	3.6	LOC256	AT3G27260
LOC256 = 1	FT = intermediate	0.722222	0.8125	1.125	LOC256	AT3G27260
LOC319 = 0	FT = late	0.111111	1	3.6	LOC319	AT3G55970
LOC319 = 1	FT = intermediate	0.722222	0.8125	1.125	LOC319	AT3G55970
LOC343 = 0	FT = intermediate	0.722222	0.8125	1.125	LOC343	AT4G06566
LOC343 = 1	FT = late	0.111111	1	3.6	LOC343	AT4G06566
LOC384 = 0	FT = intermediate	0.722222	0.866667	1.2	LOC384	AT4G17330
LOC384 = 1	FT = late	0.166667	1	3.6	LOC384	AT4G17330
LOC390 = 0	FT = intermediate	0.722222	0.8125	1.125	LOC390	AT4G20510
LOC390 = 1	FT = late	0.111111	1	3.6	LOC390	AT4G20510
LOC416 = 0	FT = intermediate	0.722222	0.8125	1.125	LOC416	AT5G18060
LOC416 = 1	FT = late	0.111111	1	3.6	LOC416	AT5G18060
LOC458 = 0	FT = intermediate	0.722222	0.8125	1.125	LOC458	AT5G37230
LOC458 = 1	FT = late	0.111111	1	3.6	LOC458	AT5G37230

The overview of the *Helitron* distributions of these ecotypes showed that *Helitron* distributions were similar in these genomes, despite the existence of minor variations among different ecotypes (Additional file 1: Figure S8). These 18 ecotypes of *Arabidopsis* genomes are of different lengths; thus, we cannot use their own physical locations for direct comparison. Therefore, the nearest gene downstream of the *Helitron* 3′ termini was used as a marker to represent the *Helitron*-inserted loci (Additional file 2: Table S2). A total of 562 loci with markers were found, and 508 of them had polymorphisms (named LOC001–508). All ecotypes sheared 54 loci and owned their unique loci (counted 1 to 14, Table 5), indicating that many active *Helitron* transpose events occurred in these ecotypes (Additional file 2: Table S2).

**Fig. 7:**
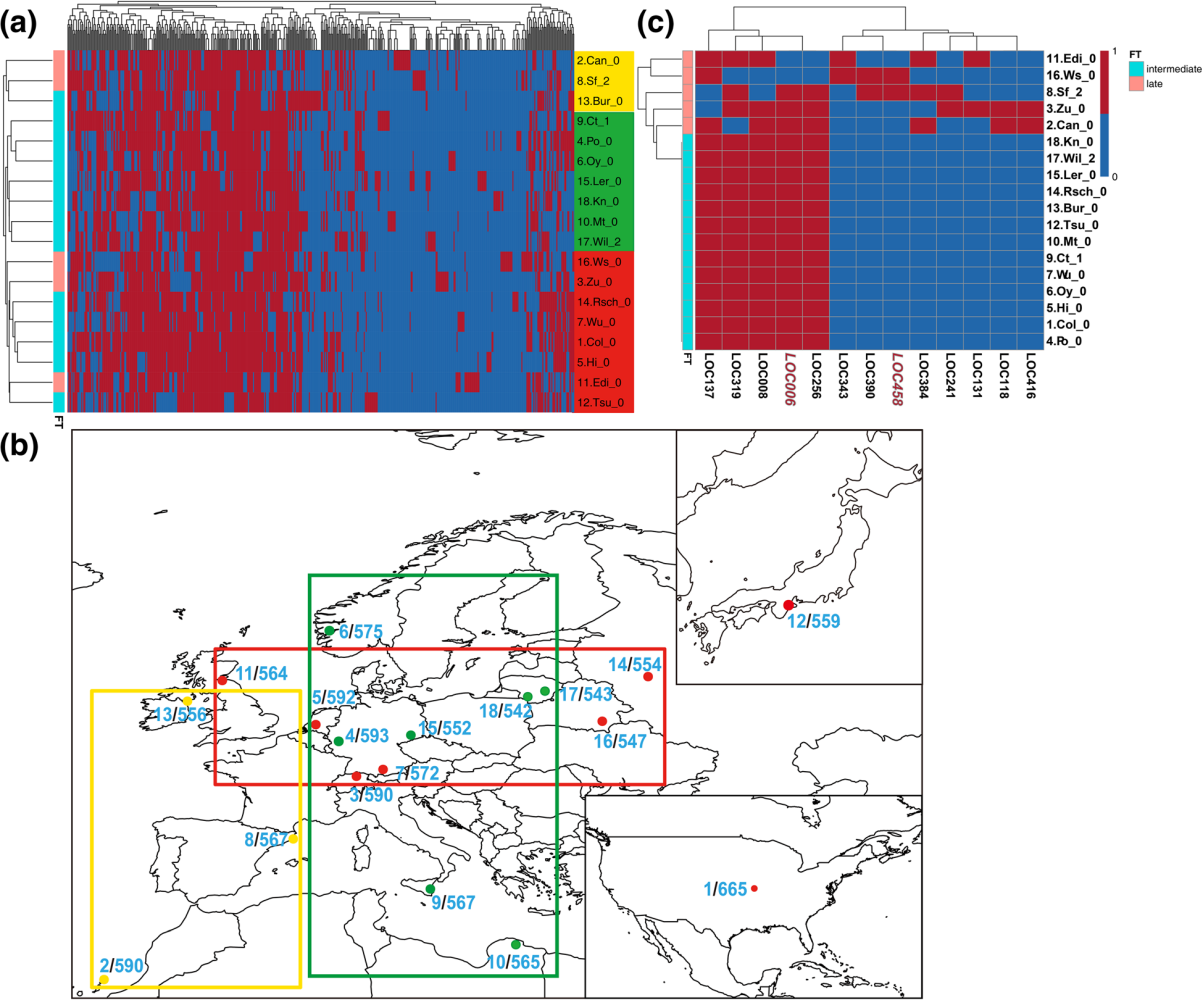
*Helitron* inserted LOCs diversity and geographical distribution of 18 *A. thaliana* ecotypes. **a** *Helitron* inserted LOCs diversity heatmap and cluster tree, red or blue means that the LOC has a *Helitron* insertion or not. Left cluster tree is mainly in three groups (yellow, green and red). **b** Geographical distribution of 18 *A. thaliana* ecotypes painted by rworldmap. Country boundaries are derived from version 1.4.0 of Natural Earth data 1:110 m data (http://www.naturalearthdata.com/downloads/110m-cultural-vectors/110m-admin-0-countries/). The number marks are *Helitron* density ranking and predicted *Helitron* numbers. Yellow, green and red points are three clustered groups. **c** Heatmap of 13 paired rules of *Helitron* LOCs that associated with flowering-time types

To investigate whether these *Helitron* distributions informed the relationship between these 18 *A. thaliana* ecotypes, we used the *Helitron* diversity LOCs matrix (Additional file 2: Table S2) and clustered the ecotypes into three main groups (Fig. 7a). Considering their geo-location information (Additional file 2: Table S2), these groups presented some aggregation of geographical distribution (Fig. 7b). The yellow group (Can-0, Sf-2, and Bur-0) in Fig. 7b, was distributed in Western and Southern Europe, and North Africa (longitude, − 13.48 to 3.03); the green group (Ct-1, Po-0, Oy-0, Ler-0, Kn-0, Mt-0, and Wil-2) was distributed in Northern, Central, Southern, and Eastern Europe, and North Africa (longitude, 6.19 to 25; latitude, 32.3 to 60.38); the red group (Ws-0, Zu-0, Hi-0, Wu-0, Rsch-0, Edi-0, except for Tsu-0 of Japan and Col-0 from the USA) were distributed in Western, Central, and Eastern Europe (longitude, − 3.16 to 34; latitude, 47.37 to 56.3). The green group was more widely distributed south-north, whereas the red group was more distributed east-west. The locations of these groups probably indicated the main direction of the spread of *A. thaliana* subgroups, and the *Helitron* polymorphism were probably correlated with the adaptation of each ecotypes to its ecological conditions.

To gain insight into the possible effect of *Helitrons*, we performed an associate analysis between the polymorphisms of *Helitrons* and flowering-time type (late type and intermediate type) in 18 ecotypes (Table 5). We found 216 single associating rules (Additional file 2: Table S11) among 13 LOC-paired rules (both 0 and 1) associated with flowing-time type (Table 7, Fig. 7c). Ten genes in upstream and downstream regions of the LOCs were searched with 306 known flowering-time related genes in *Arabidopsis* [42]. We found that two *Helitron* polymorphism loci, LOC006 (near AT1G04425) and LOC458 (near AT5G37230), which belonged to the 13 paired rules, were closely linked with two flowering-related genes Cryptochrome-2 (*CRY2*, AT1G04400) [43] and Circadian 1/Reveille 2 (*CIR1/RVE2*, AT5G37260) [44].

**Fig. 8:**
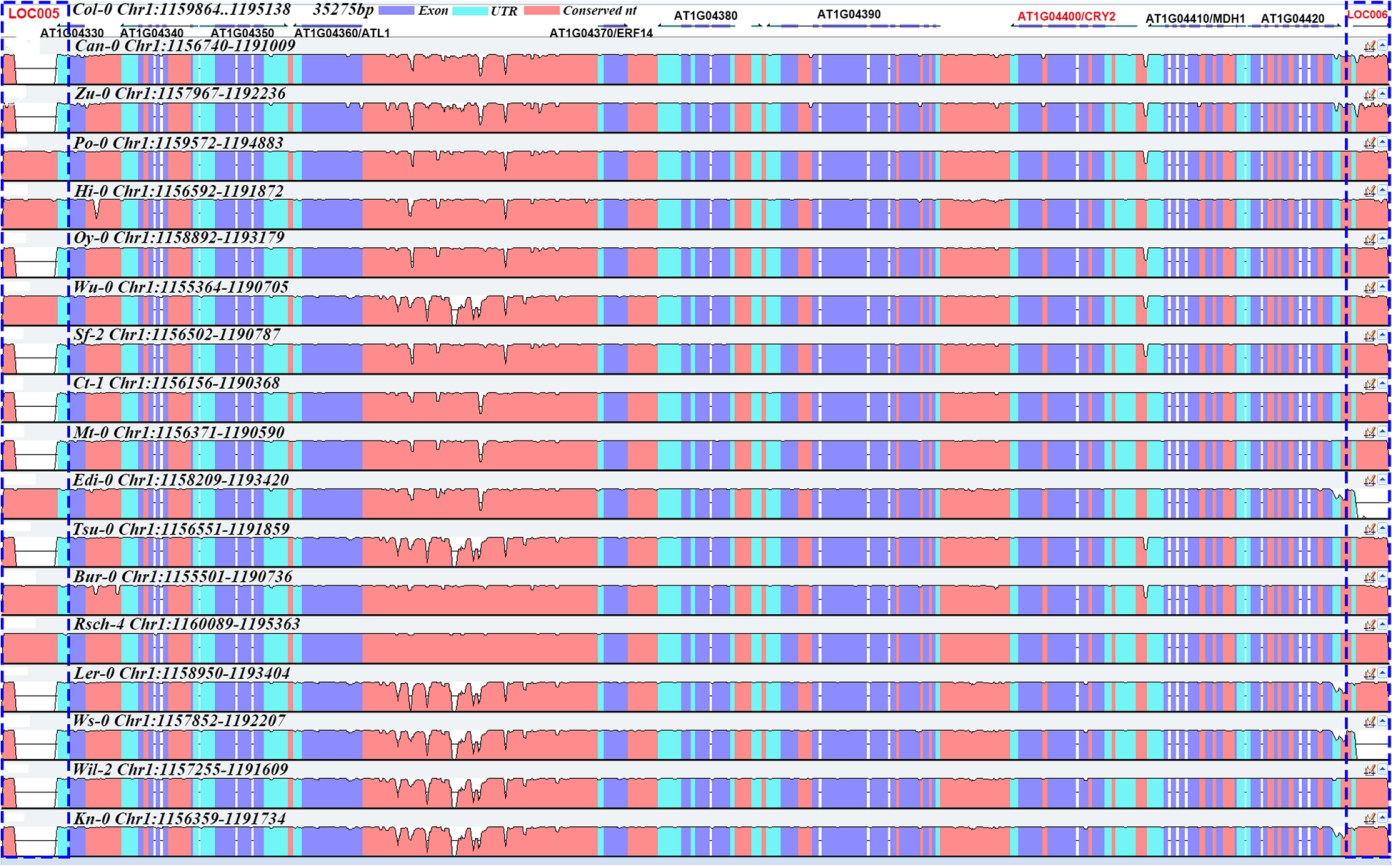
VISTA plot of sequence difference of 18 *A. thaliana* ecotypes (LOC005 to LOC006). The first row is gene annotation of Col-0 as reference. Blue means exon part, light blue means UTR, dark red means conserved sequence. Flowering related gene *CRY2* (AT1G04400) that near the LOC006 is associated with flowering-time phenotype by association analysis

To confirm the *Helitron* LOC diversity of these ecotypes, we cut the LOC006 part of the sequences of all 18 genomes, and the resulting VISTA plot of the LOC005 to LOC006 multiple sequence analysis is presented in Fig. 8. It shows that these two LOCs have different sequence lengths in these ecotypes (same as Fig. 7c and Additional file 2: Table S3) and that the flowering-related gene *CRY2* (AT1G04400) near LOC006 (near AT1G04425) was only two genes away. The analysis might suggest a correlation between *Helitron* insertion and flowering-time phenotypes, and that *Helitron* polymorphisms may be informative for association studies.

## Discussion

Historically, research has focused on the protein-coding genes, and the rest of the genome was considered to be ‘junk DNA’. Recently, it has been shown in multiple studies that these ‘junk’ regions affect biological processes involving miRNA, LncRNA, and TEs [45]. *Helitron*s are unique rolling-cycle type transposons for which like other transposons, their activation may involve the generation of new genes or regulating existing genes, and potentially affecting phenotype expression [46]. Researching non-coding sequences, such as *Helitron*s, is an important task as well as studying protein sequences. Here, we provide another effective toolkit (EAHelitron and related scripts) for researchers to annotate *Helitrons* in sequenced genomes. EAHelitron was found to be fast and was able to find many new *Helitron*s that were not predicted by other programs. With the output GFF files, it was easy to visualize the *Helitron* locations, terminal ends and flanking sequences, and are useful for further study, such as insertion annotation, transposon classification, captured gene identification, model contracture, etc. EAHelitron can expediate the annotation of *Helitrons* in the rapidly increasing number of sequenced plant genomes.

Previous research found that most of *Helitrons* are relatively young with 87% of Basho elements to have originated in the last 5 Myr. This was subsequent to the divergence of *A. thaliana* from its closest relative *A. lyrata* [47], that occurred about 10 Mya [41]. Our *Helitron* homology rate analysis of these two close species also supported a young age of most *Helitrons* (Additional file 2: Table S6). Random sequences analysis for FPR also found that, novel classical terminal ends are not difficult during genome recombination. It suggested that de novo *Helitron* ends near the RepHel motif were probably a source of younger *Helitron*s or lineage-specific *Helitron*s in a species.

**Fig. 9:**
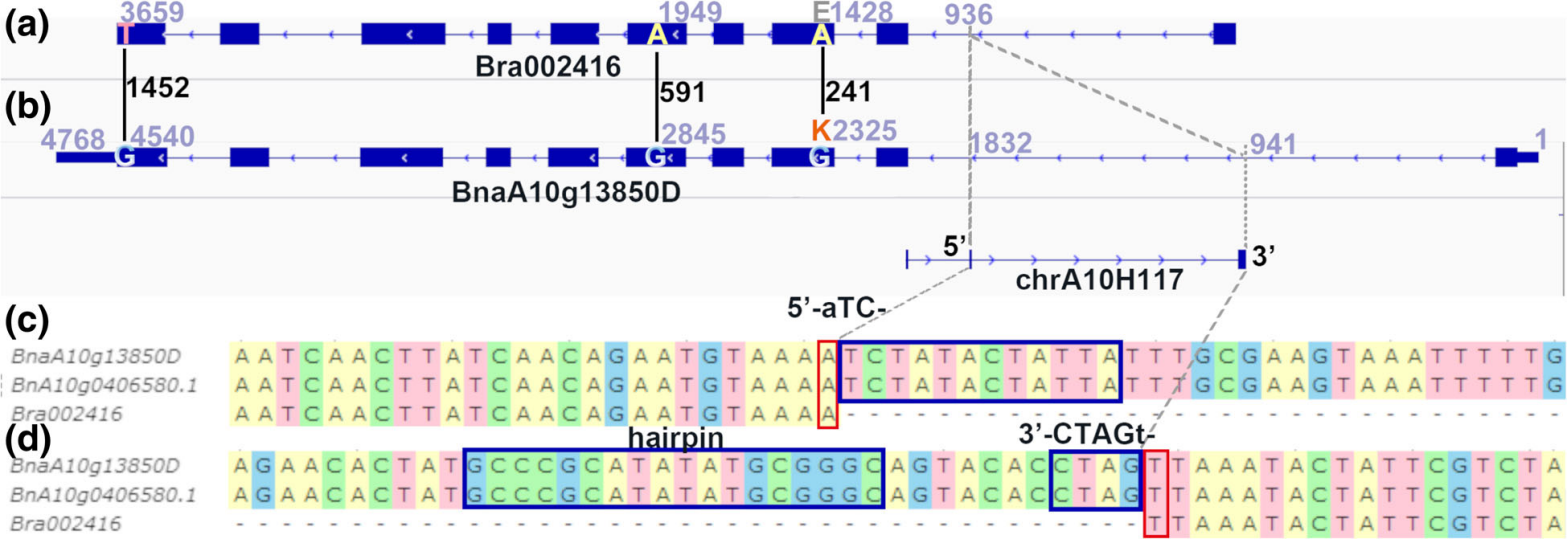
Compare with the ancient gene, a *Helitron* is inserted into the first intron of BnaA10g13850D of *B. napus*. **a** Homologous ancient gene Bra002416 of *B. rapa*, no *Helitron* predicted in the second row. **b** EAHelitron annotated *Helitron* chrA10H117 in the first intron of BnaA10g13850D (second row), form 941 to 1832 (red number using 3′-UTR as start), these homologous genes had three SNPs in CDS, in 241(A/G), 591(A/G) and 145(T/G) (black number using CDS start codon as 1), only the first SNP changes the translation from E (glutamic acid) to K (lysine). **c** *Helitron* 5′-TC terminal. **d** *Helitron* 3′ hairpin and CTAG end. The *Helitron* is inserted into an -AT- site

*Helitron*s are important players in the evolution of plant genomes, and therefore genome location information may be useful for future research. Many important species in Brassicaceae have had their whole genome sequenced and are therefore useful for studying the evolution and domestication of these plants. This study noted the distribution and classification of *Helitrons* predicted in the Brassicaceae genomes. We found that *Helitrons* like other TEs, accumulate in gene-poor regions [47], and insertions tend not to directly insert into coding regions because most of these events are detrimental to the gene and are therefore selected against. The Brassicaceae *Helitron*s were concentrated near the centromeres, the regions with low recombination rates, like *A. thaliana* [18]. Moreover, some *Helitrons* might be correlated with some domestication traces in genomes. Previous researches showed that, *Helitron* insertions affect seed coat color of *B. rapa* [26] and self-compatibility of *B. napus* [27]. In our study, *Helitron*-inserted genes in *B. rapa* were enriched in water transmembrane transport functions, which might explain the high water content of Chinese cabbage (Additional file 5: Table S9). Besides, *Helitron-*inserted genes in *B. napus* were enriched in long-chain-fatty-acyl-CoA reductase activity (BnaA10g13850D, BnaC02g09500D, BnaCnng48650D, BnaCnng47950D, and BnaCnng48640D) and other long-chain-fatty-related terms (map00061: Fatty acid biosynthesis, Additional file 5: Table S9), thereby indicating that *Helitrons* might have contributed to the natural variation of lipid quality during the domestication of *B. napus*. We took BnaA10g13850D of *B. napus* Darmor as an example, which was a fatty acid reductase 1 (*FAR1*, AT5G22500) homologous gene involved in oxidoreductase activity, fatty-acyl-CoA reductase (alcohol-forming) activity (Fig. 9a, b). The genomic sequence of BnaA10g13850D was 4768 bp from 5′ UTR to 3′ UTR (chrA10:11115015–11,119,782, minus strand). An 892-bp *Helitron* insertion annotated as chrA10H117 in *B. napus* by EAHelitron, was observed in the first intron (941–1832) of BnaA10g13850D of *B. napus*, compared with its ancestral gene Bra002416 in *B. rapa* (3884 bp, A10: 9784847–9,788,730, minus strand). Double low oilseed rape cultivar has long been the objective of domestication. *Brassica napus* Darmor is a French winter double low oilseed rape cultivar, which lacks detectable erucic acid in the seed oil with low seed glucosinolate content [38]. *Brassica napus* Zhongshang11 (ZS11), an Asian semi-winter oilseed rape cultivar, is also a double low oilseed rape cultivar [48]. This cultivar ZS11 also contained *Helitron* chrA10H117 in the paralogous gene of BnaA10g13850D (Fig. 9 c, d), suggesting that *Helitron* insertions may had contributed to the lipid quality to a certain extent in the domestication of *B. napus*. These *Helitron* insertions’ effects need further research. These results imply that humans probably have selected the genomic variation caused by *Helitron* insertions. These *Helitron* insertions of Brassicaceae can be useful for future genetics and molecular breeding selection studies.

Regarding the 18 different ecotype genomes of *A. thaliana*, we found at least 508 active *Helitron* suggesting *Helitron*-insertion polymorphisms. Comparing the genomic differences between multiple ecotypes of a species, combined with geographic and environmental information may contribute to the study of species diversity history. And we get similar spread directions of European *A. thaliana* after the ice retreated (east to west, south to north), which previous research reported, starting ~ 10,000 years ago [49]. This study attempted to use *Helitron* LOC diversity to correlate the phenotypes of the *A. thaliana* ecotypes in terms of flowering time, and we obtained 13 associated locations, including two LOCs near known flowering-related genes. We need further research to determine these *Helitron* insertions’ function. It suggested that *Helitron* polymorphisms have the potential applicability in genome wide association studies (GWAS) as a bio-marker, similar to SNP/indel and copy number variation, which will help in improving GWAS maker numbers in samples. In addition, because of the many single-copy *Helitron*s present in *Arabidopsis*, information regarding the positional variation of single-copy *Helitrons* between different ecotypes is informative for studying the *Helitron* ‘cut-and-paste’ transposition mechanism.

We annotated thousands of *Helitron*s in the genomes of 53 plants including monocots and eudicots. We did not observe a significant difference between monocots and eudicots, and they both had wide ranges of *Helitron* abundance and genome size. In Xiong’s research, they found no sign of correlation between *Helitron* abundance and genome size [4]. In contrast, our study showed that *Helitron* abundance is positively correlated with genome size suggested that *Helitrons*, like other TEs, contribute to changes in genome size [46].

*Helitron* 5′ terminal ends are not as conserved as the 3′ ends, and a *Helitron* may have multiple 5′ ends [4], so the predicted full lengths of the *Helitrons* might not be accurate. The 3′ ends with their hairpins played important roles as a transposition terminator [5], thus the number of 3′ ends could be a base of a minimum number of total *Helitron* [18]. *Helitron* density, calculated using *Helitron* number and genome size, could be as a more accurate characteristic. We did not find any significant *Helitron* density related patterns between monocots and eudicots. However, we did find that many closely related species have more changes in *Helitron* density. As many *Helitrons* use a ‘cut and paste’ mechanism, their copy numbers remain low. Genome sequencing and annotation methods might have little effect on the result. For instance, the genome sequence of maize “B73” AGPv3 (6a) was produced by the Maize Genome Sequencing Project, and the alignment of “Mo17” 454 reads to this reference sequence, finally assembled a 2067 Mbp genome (Fig. 3). An entirely new assembly of the maize genome (B73 RefGen_v4, 2134 Mbp) was further constructed from PacBio Single Molecule Real-Time (SMRT) sequencing, and the genome size of such new version became bigger, but the *Helitron* number reduced (8622 and 8274, respectively, Table 3, Fig. 3). It was likely the longer reads based on 3GS technology lead to reduce number of repeat sequences in final assembled genomes. In an analysis of seven multiply sequenced genomes, we found that *Helitron* density is probably stable at species level. We also found that *Helitron* density was not correlated with genome size, which suggested that *Helitron* density is independent from genome size. Based on these results, we consider that *Helitron* density has potential applicability in species classification. We used de novo assembled scaffolds from NGS data of two *A. thaliana* T-DNA mutants to determine genome size and *Helitron* density, then used LDA to identify its species. The two test samples were successfully identified as *A. thaliana*. Therefore, with the development of new sequencing technology, the *Helitron* density could be considered as a quick way to identify an unknown plant sample.

The first Angiosperm Phylogeny Group (APG I) classification of the orders and families of flowering plants is a modern molecular-based system of plant taxonomy, which is based on the cladistic analysis of the DNA sequences of two plastid/chloroplast genes (*rbc*L, *atp*B) and one gene of ribosomes (18S rDNA) [37]. Although it is based only on molecular evidence, its constituent groups have been further supported by other morphology and chemistry evidence as well. For example, pollen feature supports the split between the eudicots and the rest of the former dicotyledons [50]. The characteristics of *Helitrons* discussed herein provide genome-scale characteristics which can bolster these classifications. A combination of *Helitron* density and other plant characters, analyzed with modern machine-learning algorithms, such as artificial neural networks, may be informative for constructing a more accurate phylogenetic tree of plant diversity. We attempted to combine the number of chromosomes with *Helitron* information. However, the same species may have multiple karyotypes of chromosome numbers, not simply related to genomic features, and so no reliable results were obtained. As further species of plants have their genomes sequenced, *Helitron*-related features could be employed to study, e.g., the difference between angiosperms and gymnosperms, herbs and woody plants, field and horticultural crops, monocots and eudicots, wild species and domesticated species, adaptation to the environment, etc.

*Helitron* density may not only represent the tolerance of the host genome to them but may also represent the rate of recombination or self-fertilizing rate of the species. According to previous reports, high self-fertilizing rates reduce the importance of recombination rates on genome structure [19]. In an outcrossing species, new TEs can spread rapidly through a population by recombination via sexual reproduction. In contrast, in self-fertilizing species, recombination is not effective at spreading TEs. New copies are therefore lost by genetic drift and/or purifying selection, and the probability of TE fixation is reduced. This would result in a lower number of new TEs copies in self-fertilizing species [51]. In this study, we compared the *Helitron* density of *B. napus* and its ancestors, *B. rapa* and *B. oleracea*, and found that the *Helitron* density of the *B. napus* subgenome was lower than that of the ancestral genomes, which may be because the ancestors were both self-incompatible. In this scenario, *B. napus* have become self-compatible following allopolyploidy in the last 7000 years. Moreover, EAHelitron could probably be applied to animals or other genomes. Also, it can be easily rewritten to search for other genome-wide features, e.g., to find other TEs or tandems, or to predict the editable site of the CRISPR/Cas gene editing system, and SSR.

## Conclusions

We developed EAHelitron, which is a fast and efficient tool to identify new *Helitrons*. Whole genome *Helitron* density can be an informative character for plant classification. We predicted thousands of *Helitrons* in Brassicaceae, *Helitron* distribution patterns of most species in this family were similar to *A. thaliana*. *Helitron* insertion polymorphism could be used in genome wide association studies. This research may contribute to speed up our research of *Helitrons* and understand their role in plant evolution.

## Methods

### Extraction of plant genome sequences and phylogenetic data

Genome sequences of 40 plants were downloaded from Phytozome version 11 (https://phytozome.jgi.doe.gov). Sequences of 16 Brassicaceae species genomes were downloaded from BARD (http://brassicadb.org/brad) and Ensemble Plant (https://plants.ensembl.org). Eighteen ecotypes of *A. thaliana* were analyzed, and their genomes sequences were downloaded from 1001 Genomes Project (http://1001genomes.org) [52]. Detailed source information for all genome sequences is listed in Additional file 3: Table S4. The phylogenetic trees of 45 genomes were also obtained from Phytozome version 11, and they were edited in TreeGraph2 [53].

### Genome sequencing and de novo assembly of LDA samples

Whole genome sequencing of two accessions of mutant *A. thaliana* SALK_015201 and CS852557, were sequenced by Illumina HiSeq 4000, and a total of 5.7 GB and 9 GB 150-bp paired-end reads sequence data were obtained. Sequence Read Archive IDs are SRR5249176 and SRR5249156. Raw data were cleaned by Trimmomatic [54]. These two de novo draft genomes were assembled using SOAPdenovo 2.40 [55], with kmer values of 81 or 85, named Denovo_genome_X (120.2 MB, N50: 25,619, scaffolds: 9888) and Denovo_genome_L (121.1 MB, N50: 5064, scaffolds: 3350).

### Performance testing of EAHelitron

The predicted results from EAHelitron are compared with those from other programs including HelSearch, HelitronScanner and RepeatMasker, based on the genome sequence of *A. thaliana* (TAIR10), a model plant of dicot species. Running time cost was also taken into account when assessing software efficacy. Venn diagram of these results were plotted in jvenn [56]. To estimate the false positive rates (FPR), 100 randomized genomes were created by shuffling the genome sequence of *A. thaliana* (TAIR10) using RandomDNA_rate.pl (A:T:G:C = 0.319414:0.319033:0.179905:0.180095; length = 119,667,750 bp, counted by CountATGC.pl). *Helitron* predictions on these randomized genomes are regarded as false positives [18]. The basic version and multi-threading version of EAHelitron, manuals and other assistant scripts are available at GitHub (https://github.com/dontkme/EAHelitron).

### *Helitron* visualization and annotation

Using the GFF files extracted from EAHelitron, the *Helitrons* and *Helitron* inserted genes were exported to an integrative genomic viewer (IGV) [34]. The output files containing the 3′ terminal ends (*.3.txt) were used to count the number of *Helitrons*, and *Helitron* density of the whole genome was defined as the number of *Helitrons* per Mb. To identify the autonomous *Helitrons* in Brassicaceae, 20 kb upstream sequence of the 3′ ends of the *Helitrons* were aligned with known RepHel protein domains in *A. thaliana* and *O. sativa* using BLASTX (evalue <1e-5). The sequences (1 kb) upstream of the 3′ end of the *Helitrons* were cut and filtered by Cutfa script (no N and exactly 1000 bp sequences). Then BLASTN (evalue <1e-5, qcov > 55, s_end > 950) was used to search for homologous *Helitron* sequences. *Helitron*-inserted locations were clustered using the CompareGFF script, and the GFF from EAHelitron was compared with the general transfer format of its official genome. The functions of the inserted genes were annotated by Blast2GO [57], and the results were combined using WEGO [58].

A heatmap of Gene Ontology (GO) terms was plotted using the pheatmap [59] package in R version 3.3.3 [60]. GO and KEGG pathway enrichment analyses were carried out using TBtools (https://github.com/CJ-Chen/TBtools/, *P* < 0.001, corrected *P* < 0.1 and hit genes > 2) [61]. The multiple sequences alignment was carried out in MUSCLE [62] and UGENE [63]. The presence or absence of LOC005 and LOC006 was visualized in VISTA [64].

### Data analysis

Statistical calculations and graph plotting were performed using R version 3.3.3 [60]. We used the cor.test function for Pearson’s product-moment correlation, boot [65] package for bootstrapped *p* values and r (1000 replicates, seed: 1234). Chisq.test function for chi-squared test. Linear discriminant analysis (LDA) was used to estimate the capability of *Helitron* density as a characteristic in plant system classification. Genome sequences of 34 varieties from seven species groups were trained using lda function of MASS package, and two de novo genomes were added as test samples [66]. For hierarchical clustering, hclust function with ‘median’ method was used to draw the Brassicaceae dendrogram. Using the SWcount script, sliding window analysis (window = 1 Mb, step = 500 kb) was carried out to investigate the local scale density of *Helitron* and genes.

To investigate the distribution of *Helitrons* in different ecotypes of *A. thaliana*, we selected the seeds of 18 ecotypes from different countries in the Arabidopsis Biological Resource Center. After 45 days of growing, the seedlings were photographed and their flowering-time types were recorded (Table 5). To identify the nearest genes of each *Helitron* as markers, 500 bp sequences downstream of *Helitrons* were BLASTNed against *Arabidopsis* TAIR10 (Col-0) coding sequence (CDS) (evalue <1e-5, q_star < 450). The genes showing similarity obtained from BLASTing and these polymorphisms loci were named LOC001 to LOC508, and were clustered in R. Geographical distribution of 18 *A. thaliana* ecotypes were painted by rworldmap [67], country boundaries were derived from version 1.4.0 of Natural Earth data 1:110 m data (http://www.naturalearthdata.com/downloads/110m-cultural-vectors/110m-admin-0-countries/). Association analysis between the *Helitron* insertion polymorphisms and flowering-time types in 18 ecotypes was performed, using the apriori function of the arules package (maxlen = 3, support > 0.1, confidence > 0.8, lift > 1.1) [68].

## Additional files

Additional file 1: **Figure S1.** TAIR10 predicted *Helitrons* from RepeatMasker and EAHelitron in IGV. **Figure S2.** Correlation of genome size, *Helitron* number and *Helitron* density of 51 genomes (exclude maize and *B. napus*). **Figure S3.** Dot plot of LDA samples. **Figure S4.** Dot plot of genome size and *Helitron* densities of 16 Brassicaceae genomes. **Figure S5.** Brassicaceae phylogenetic trees. **Figure S6. ***Helitron* insertion examples in IGV. **Figure S7.** The 45 day live plant photos of 18 *A. thaliana* ecotypes. **Figure S8. ***Helitron* distribution of 18 *A. thaliana* ecotype genomes. (PPTX 6583 kb)

Additional file 2: **Table S1.** EAHelitron uniquely predicted* Helitrons* and polymorphisms loci. **Table S2.** All *Helitron* LOCs and nearest TAIR ID of 18 Ath ecotypes with phenotypes. **Table S3.** False positive rate of *Helitron* programs (100 random sequences).  **Table S5.** RepHel percent of *Helitrons* in Brassicaceae. **Table S6.** Six Brassicaceae genomes *Helitron* 3’ end upstream 1 kbp sequences homology rate. **Table S7.** Brassicaceae gene density correlation of *Helitron* density (window = 1 Mb, step = 500 kb). **Table S10.** TAIR10 predict *Helitron* inserted into intron or UTR genes. **Table S11.** The 216 single rules associated with Flowering-Time types. (XLSX 139 kb)

Additional file 3: **Table S4.** All Genomes information. (XLSX 34 kb)

Additional file 4: **Table S8.** Brassicaceae *Helitron* inserted genes. (XLSX 4171 kb)

Additional file 5: **Table S9.** GO and KEGG pathway enrichment of Brassicaceae *Helitron* inserted genes. (XLSX 33 kb)

## Data Availability

The scripts developed as part of the study are available at GitHub (https://github.com/dontkme/EAHelitron). The raw data of two accessions of mutant *A. thaliana* SALK_015201 and CS852557, was deposited at NCBI SRA database under accession SRR5249176 and SRR5249156. All *A. thaliana* seeds are ordered from Arabidopsis Biological Resource Center (ABRC). Other datasets generated or analyzed during this study are included in this published article and its supplementary information files.

## Abbreviations

Aly: *Arabidopsis lyrata*
APG: Angiosperm Phylogeny Group
Ath: *Arabidopsis thliana*
Bna: *Brassica napus*
Bol: *Brassica olerracea*
Bra: *Brassica rapa*
CDS: coding sequence
Cru: *Capsella rubella*
Csa: *Camelina sativa*
FPR: False positive rates
GFF: General feature format
GO: Gene ontology
GTF: General transfer format
GWAS: Genome wide association studies
IGV: Integrative genomic viewer
LDA: Linear discriminant analysis
Mya: Million years ago
NGS: Next-generation sequencing
RCR: Rolling-circle replication
SNP: Single nucleotide polymorphism
TE: Transposable element
Tpa: *Thellungiella parvula*
Tsa: *Thellungiella salsuginea*
UTR: Untranslated region
