# Supplementary material for: Helitron distribution in Brassicaceae and whole Genome Helitron density as a character for distinguishing plant species
Source: BMC Bioinformatics. 2019 Jun 24;20:354. doi: 10.1186/s12859-019-2945-8 (PMC6591975; doi:10.1186/s12859-019-2945-8)
Supplement: Supplementary file 1 — Figure S1. TAIR10 predicted Helitrons from RepeatMasker and EAHelitron in IGV. Figure S2. Correlation of genome size, Helitron number and Helitron density of 51 genomes (exclude maize and B. napus). Figure S3. Dot plot of LDA samples. Figure S4. Dot plot of genome size and Helitron densities of 16 Brassicaceae genomes. Figure S5. Brassicaceae phylogenetic trees. Figure S6. Helitron insertion examples in IGV. Figure S7. The 45 day live plant photos of 18 A. thaliana ecotypes. Figure S8. Helitron distribution of 18 A. thaliana ecotype genomes. (PPTX 6583 kb) [file 12859_2019_2945_MOESM1_ESM.pptx]

## Slide 1
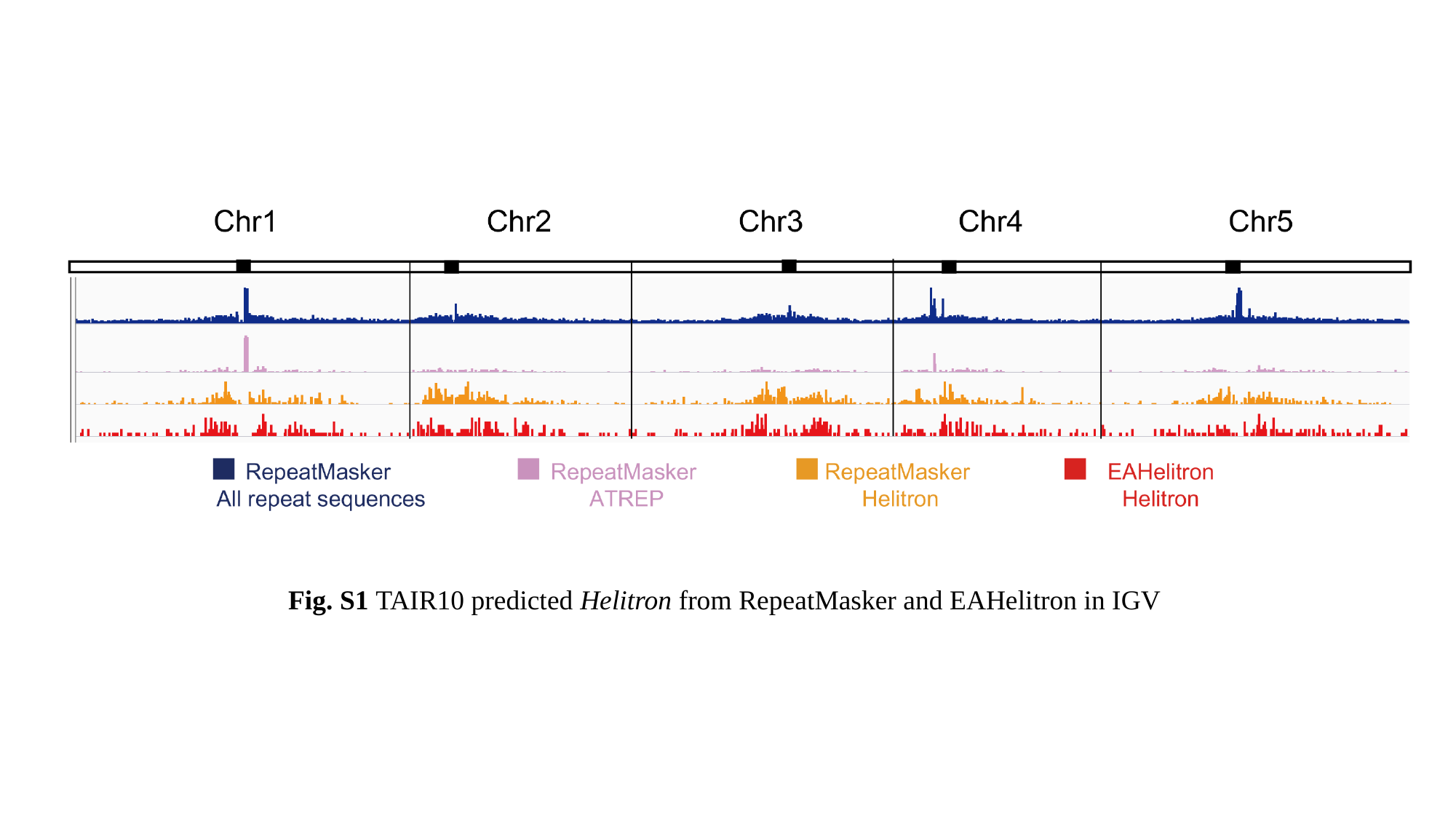

Fig. S1 TAIR10 predicted Helitron from RepeatMasker and EAHelitron in IGV

## Slide 2
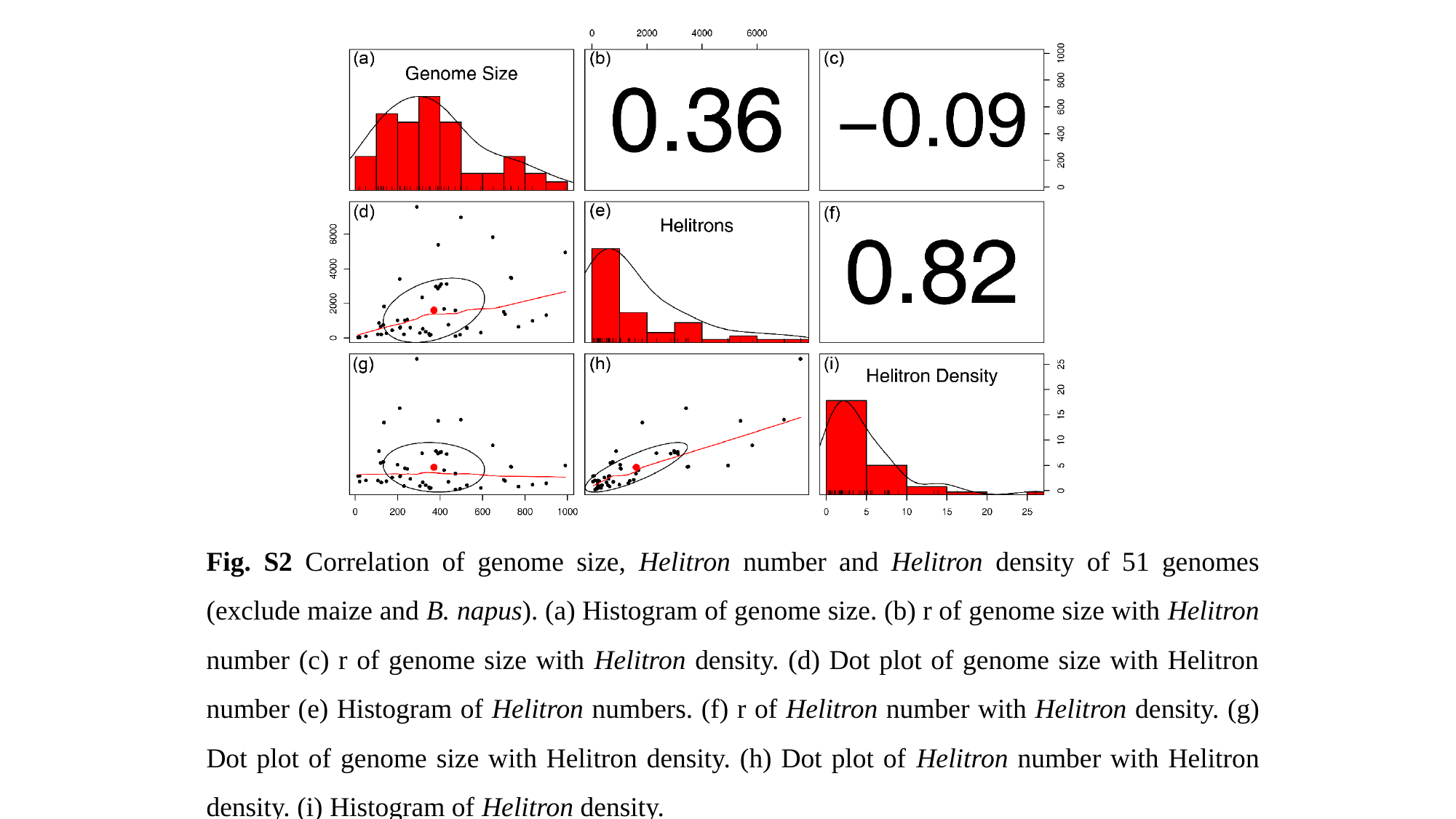

Fig. S2 Correlation of genome size, Helitron number and Helitron density of 51 genomes (exclude maize and B. napus). (a) Histogram of genome size. (b) r of genome size with Helitron number (c) r of genome size with Helitron density. (d) Dot plot of genome size with Helitron number (e) Histogram of Helitron numbers. (f) r of Helitron number with Helitron density. (g) Dot plot of genome size with Helitron density. (h) Dot plot of Helitron number with Helitron density. (i) Histogram of Helitron density.

## Slide 3
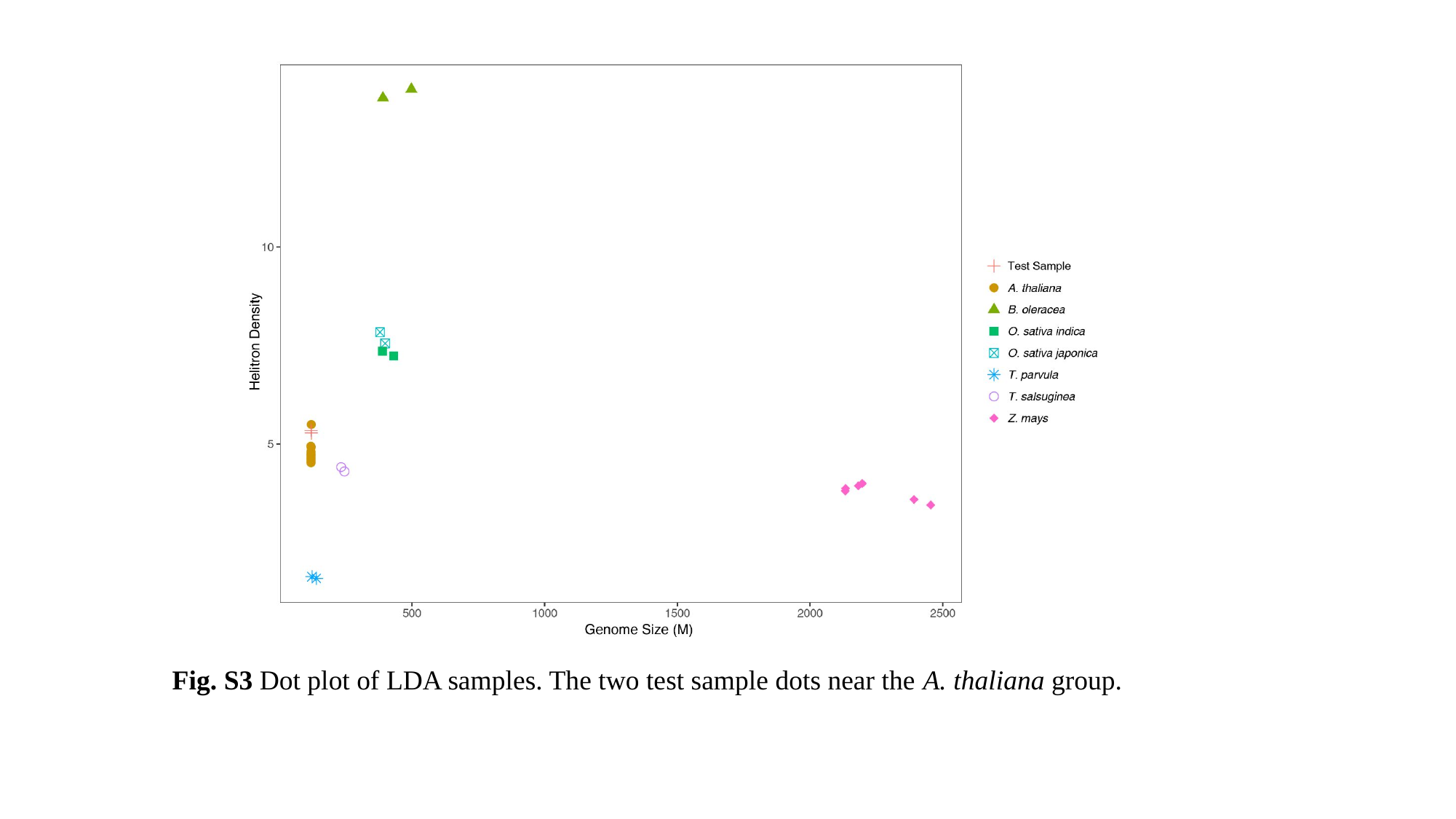

Fig. S3 Dot plot of LDA samples. The two test sample dots near the A. thaliana group.

## Slide 4
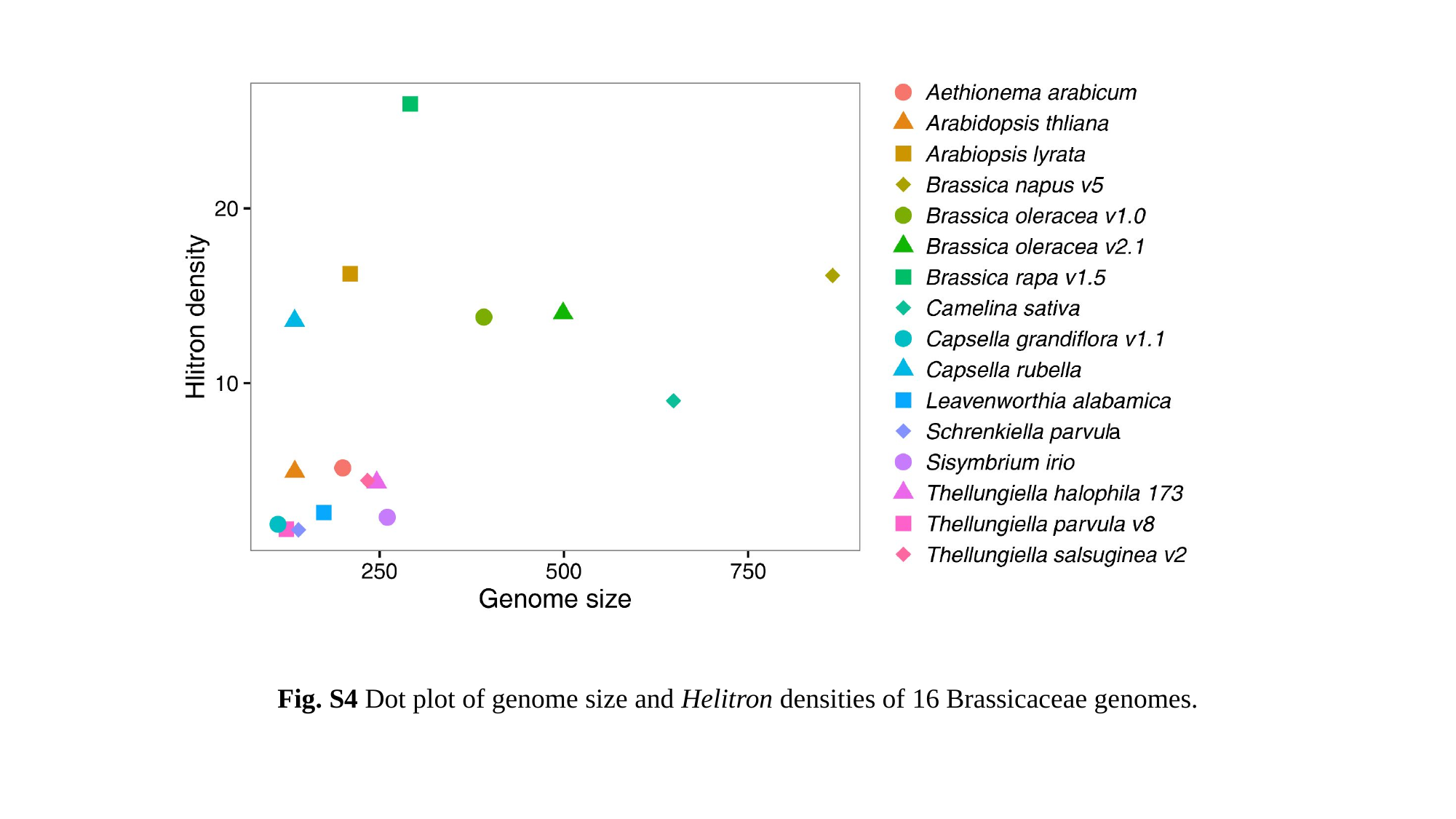

Fig. S4 Dot plot of genome size and Helitron densities of 16 Brassicaceae genomes.

## Slide 5
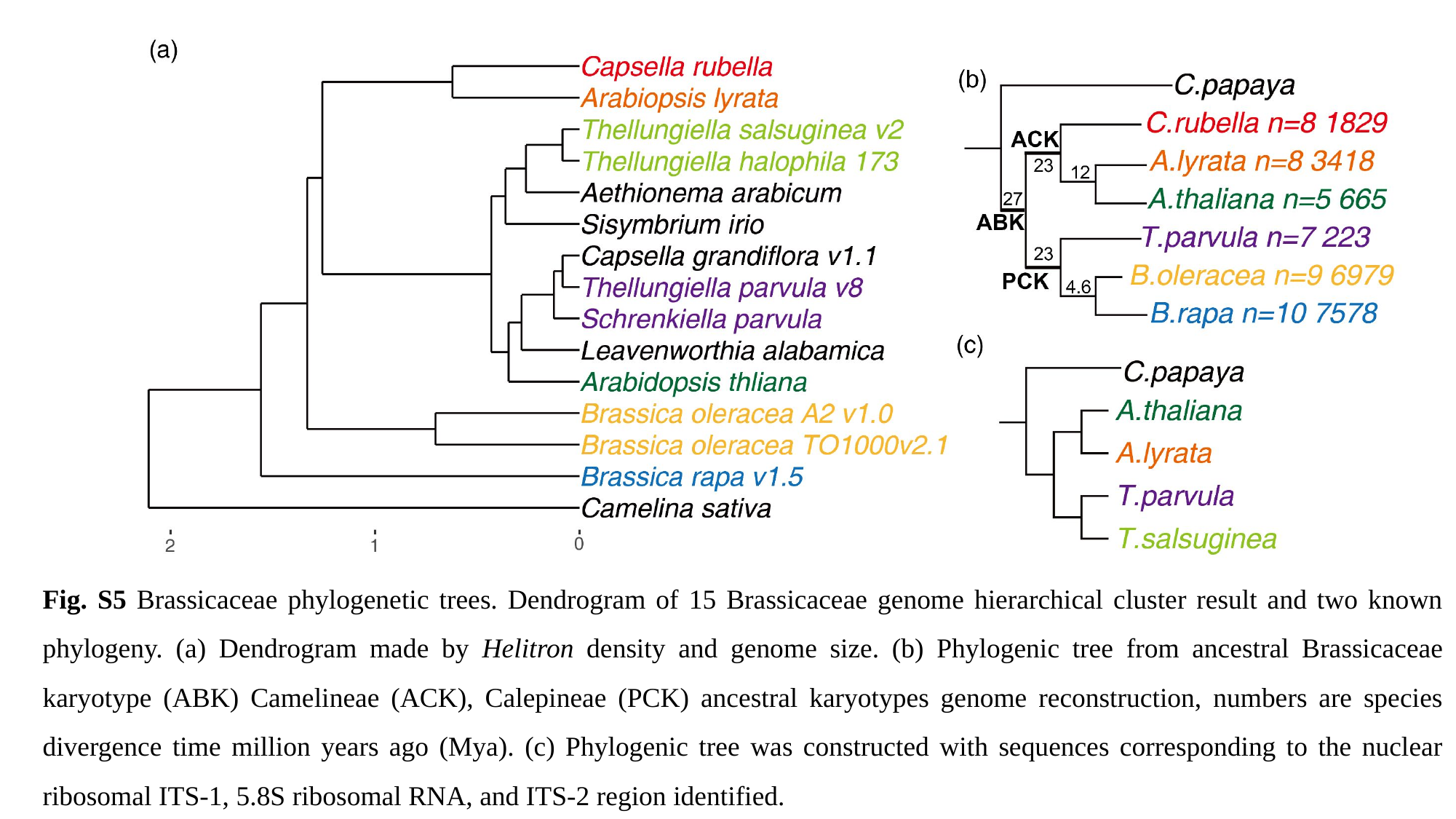

Fig. S5 Brassicaceae phylogenetic trees. Dendrogram of 15 Brassicaceae genome hierarchical cluster result and two known phylogeny. (a) Dendrogram made by Helitron density and genome size. (b) Phylogenic tree from ancestral Brassicaceae karyotype (ABK) Camelineae (ACK), Calepineae (PCK) ancestral karyotypes genome reconstruction, numbers are species divergence time million years ago (Mya). (c) Phylogenic tree was constructed with sequences corresponding to the nuclear ribosomal ITS-1, 5.8S ribosomal RNA, and ITS-2 region identified.

## Slide 6
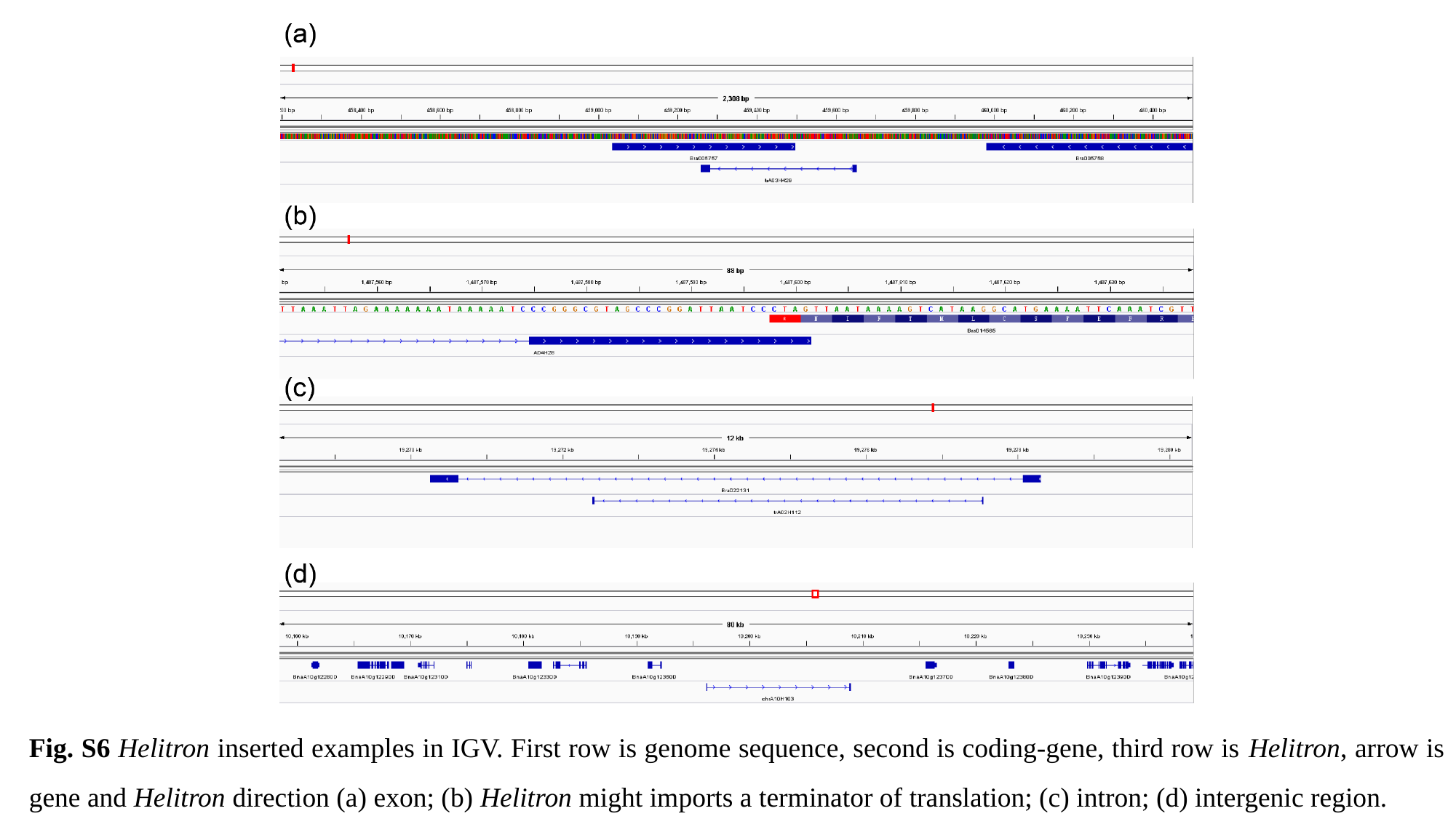

Fig. S6 Helitron inserted examples in IGV. First row is genome sequence, second is coding-gene, third row is Helitron, arrow is gene and Helitron direction (a) exon; (b) Helitron might imports a terminator of translation; (c) intron; (d) intergenic region.

## Slide 7
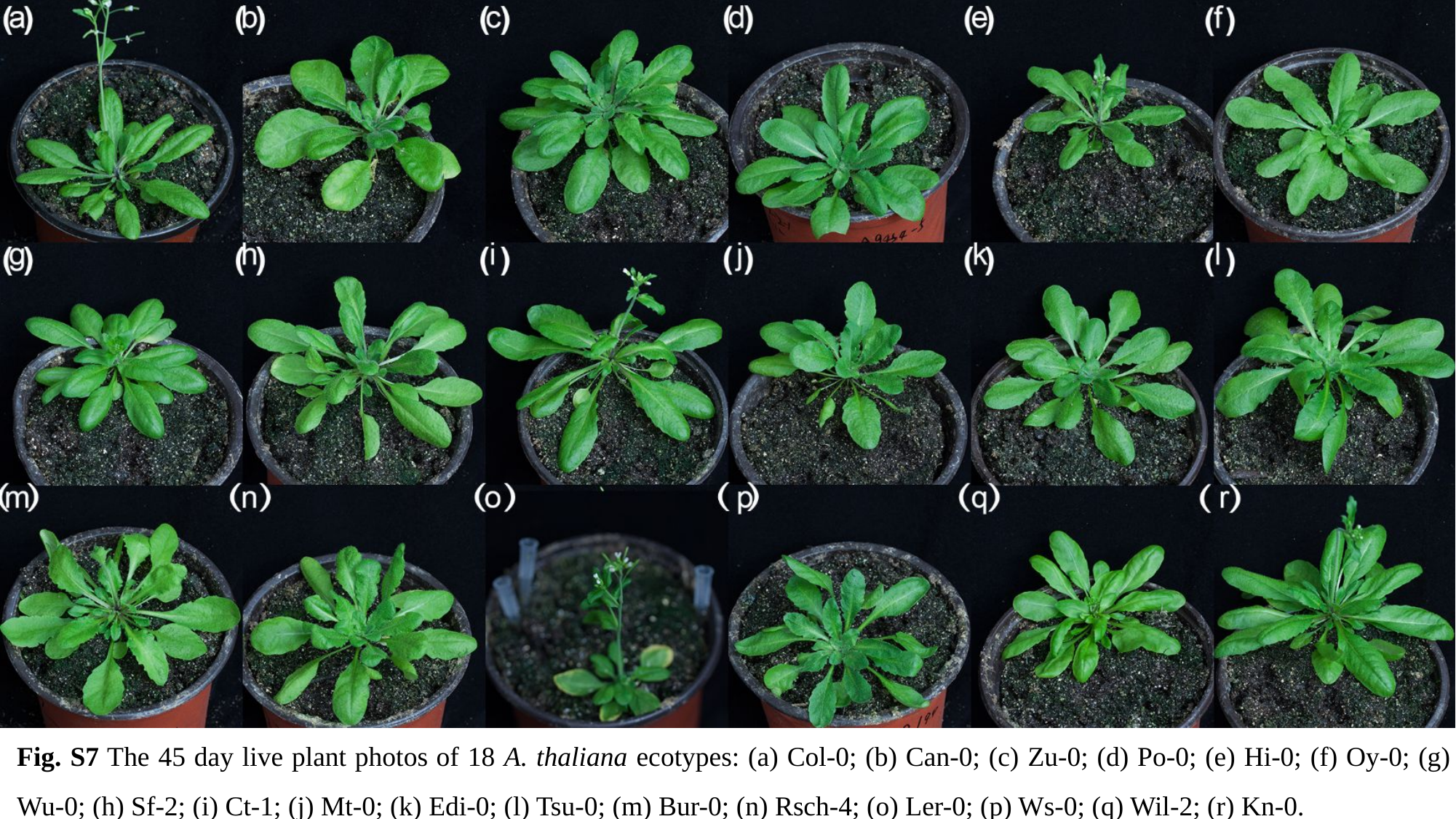

Fig. S7 The 45 day live plant photos of 18 A. thaliana ecotypes: (a) Col-0; (b) Can-0; (c) Zu-0; (d) Po-0; (e) Hi-0; (f) Oy-0; (g) Wu-0; (h) Sf-2; (i) Ct-1; (j) Mt-0; (k) Edi-0; (l) Tsu-0; (m) Bur-0; (n) Rsch-4; (o) Ler-0; (p) Ws-0; (q) Wil-2; (r) Kn-0.

## Slide 8
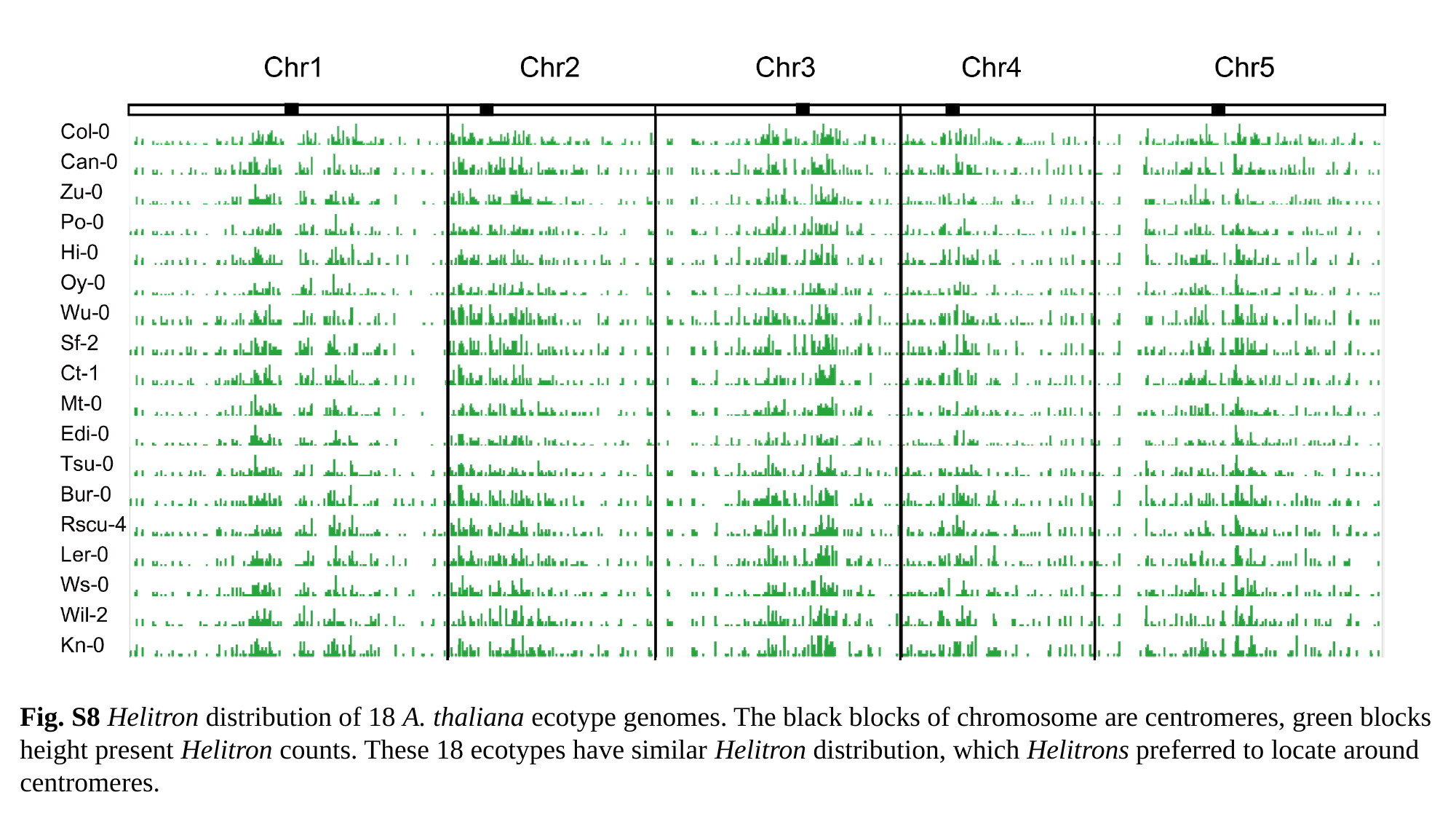

Fig. S8 Helitron distribution of 18 A. thaliana ecotype genomes. The black blocks of chromosome are centromeres, green blocks height present Helitron counts. These 18 ecotypes have similar Helitron distribution, which Helitrons preferred to locate around centromeres.
